# Mitochondrial respiratory chain regulates HBV clearance through dual modulation of lysosomal acidification

**DOI:** 10.1080/22221751.2025.2563079

**Published:** 2025-10-07

**Authors:** Zhiqiang Wei, Yanying Yan, Lingzhu Zhao, Chen Li, Jinjin Qi, Dandan Chen, Xiuzhen Huang, Minwei Li, Zhengyun Xiao, Guohua Lou, Zhenggang Yang, Mengji Lu, Xueyu Wang, Min Zheng

**Affiliations:** a State Key Laboratory for Diagnosis and Treatment of Infectious Diseases, National Clinical Research Center for Infectious Diseases, Collaborative Innovation Center for Diagnosis and Treatment of Infectious Diseases, The First Affiliated Hospital, College of Medicine, Zhejiang University, Hangzhou, People’s Republic of China; b Institute of Virology, University Hospital Essen, University of Duisburg-Essen, Essen, Germany

**Keywords:** Lysosomal degradation, hepatitis B virus, lysosomal membrane permeabilization, mtROS, mitochondrial ATP synthesis, mitochondira-lysosomal contacts, V-ATPase assembly

## Abstract

Mitochondria are vital for maintaining cellular homeostasis. However, mitochondrial damage is evident in patients with chronic hepatitis B (CHB). The role of mitochondrial dysfunction in the persistence of viral replication remains unclear. Therefore, this study aims to investigate the impact of mitochondrial dysfunction on HBV replication and elucidate the underlying mechanisms. Both mitochondria and lysosomes were dysfunctional in HBV-replicating cells. Moreover, HBV replication inhibited mitochondrial respiratory chain both *in vitro* and *in vivo*. Moderate inhibition of mitochondrial respiratory complex I activity using rotenone (Rot) increased HBV replication and decreased autophagic degradation capacity *in vitro* and *in vivo*. Mechanistically, elevated mitochondrial reactive oxygen species (mtROS) levels by Rot treatment or SOD2 knockdown led to deteriorated lysosomal membrane permeabilization, which elevated lysosomal pH and promoted HBV replication. Conversely, scavenging mtROS with mitoquinone (mitoQ) and mitoTEMPO (mitoT) had the opposite effect. Additionally, mitochondrial dysfunction reduced mitochondrial ATP production and diminished mitochondria-lysosome contacts. Obstructing mitochondrial ATP synthesis with Oligomycin A treatment or disruption of mitochondria-lysosome contacts with vacuolar protein sorting 13 A (VPS13A) knockdown resulted in lysosomal alkalinization and increased HBV replication by inhibiting vacuolar (H+)-adenosine triphosphatase (v-ATPase) assembly in an mtROS-independent manner. Ultimately, inhibition of mitochondrial complex I facilitated HBV secretion by promoting endosomal trafficking of HBV components. In conclusion, mitochondrial function plays a crucial role in HBV autophagic degradation. HBV impairs mitochondrial function, leading to a reduction in the lysosomal degradation capacity, which may hinder effective clearance of the virus.

## Introduction

Hepatitis B virus (HBV) continues to be a major global health concern, responsible for approximately 1 million deaths worldwide annually, primarily due to the progression of chronic hepatitis B (CHB) to severe liver diseases such as cirrhosis, liver failure, and hepatocellular carcinoma (HCC) [1]. Although nucleoside analogues and interferons are the primary therapeutic agents used in clinical settings, achieving complete viral clearance remains challenging. Gaining a deeper understanding of the interactions between the virus and the host can enhance our knowledge of the viral life cycle and aid in the development of new therapeutic strategies.

Mitochondria are crucial organelles involved in cellular homeostasis in both physiological and pathological conditions [2]. Mitochondrial abnormalities have been observed in the hepatocytes of CHB patients [3,4]. Additionally, numerous studies have shown that HBV infection, especially HBx expression, can cause mitochondrial dysfunction [5,6]. Given that mitochondria serve as the central hub of cellular signalling pathways, mitochondrial dysfunction inevitably results in significant alterations. However, it remains unclear whether an imbalance in mitochondrial homeostasis induced by HBV, in turn, impacts the HBV life cycle.

Autophagy is the primary pathway for clearing damaged mitochondria [7], involving the sequestration of mitochondria by autophagosomes followed by their delivery to lysosomes. Lysosomes, the main degradative compartments of eukaryotic cells, also play crucial roles in maintaining cellular homeostasis. Mitochondria and lysosomes are functionally coordinated, and the co-dysfunction of these two organelles has been observed in many chronic diseases, including chronic HBV infection and non-infectious chronic diseases such as neurodegenerative disorders and aging [8,9]. The membrane contacts between mitochondria and lysosomes also regulate mitochondrial fission [10]. In turn, mitochondrial dysfunction impacts lysosomal activity [11,12], yet the underlying mechanisms require further exploration.

Emerging evidence highlights the complex interplay between HBV and cellular autophagy. Although HBV infection triggers an initial autophagic response in hepatocytes to facilitate viral replication and assembly [13–17], lysosome-mediated autophagic degradation represents the primary pathway for spontaneous viral clearance [17–19]. However, HBV infection inhibits lysosomal activity, which in turn creates an environment for HBV replication and thus forms a vicious circle [14,20]. This vicious circle may play a key role in disease development and progression [21].

Therefore, the present study aimed to investigate whether and how mitochondrial dysfunction impacts the HBV life cycle, the important role of lysosomes in this process and the underlying mechanisms. Our findings indicate that HBV disrupts the mitochondrial respiratory chain, which in turn hampers HBV autophagic degradation by inhibiting lysosomal acidification.

## Materials and methods

### Cell culture, transfection and lentiviral infection

All the cell lines were cultured in a humidified atmosphere containing 5% CO_2_ at 37 °C. Huh7, NTCP-HepG2 and HepAD38 cells were grown in Dulbecco’s modified Eagle’s medium (DMEM), supplemented with 10% inactivated fetal bovine serum (FBS), 100 U/mL penicillin, 100 μg/mL streptomycin (Gibco), 1% nonessential amino acids (NEAA), and 1% HEPES. Primary human hepatocytes (PHHs) were cultured in primary hepatocyte maintenance medium (PMM). The siRNA/shRNA sequences are shown in Table S1. Transfection of siRNAs (40 nM) or plasmids was performed using Lipofectamine 2000 transfection reagent (Invitrogen), according to the manufacturer’s instructions. The shRNA targeting the same sequences as the siRNA was delivered into cells via lentiviral transduction. pSM2 is a 2× full-length, replication-competent HBV plasmid used for *in vitro* studies in Huh7 cells to analyze viral replication and protein expression. HBV1.3 is a 1.3× full-length HBV plasmid, also used for Huh7 cell transfection. These plasmids and their mutants were donated by Professor Mengji Lu from the Essen University Hospital. Additional plasmid sources are listed in Table S2.

### HBV mouse model and treatment

Male 6–8-week-old C57BL/6 mice were purchased from GemPharmatech Co., Ltd. (Nanjing, China) and fed with standard laboratory diet and water in a temperature-controlled pathogen-free environment. To mimic chronic HBV infection, ten micrograms of the 1.2× full-length HBV plasmid pAAV-HBV1.2 was dissolved in PBS at a volume equivalent to 10% of the mouse’s body weight and hydrodynamically injected into the tail vein within 5–7 s, resulting in stable and long-term HBV expression [22]. One week after injection, the mice were gavaged with CMC-Na (Ctrl) or rotenone (Rot, 8 mg/kg body weight) daily for 2 weeks. Serum and liver samples were collected at indicated the time points. Male AAV-HBV mice were generated by transducing with 1×10^11^ viral genome equivalents through the tail vein. Liver samples from the mice were collected at 4 weeks after transduction. All animal experimental protocols were approved by the Ethics Review and Scientific Investigation Committee of the First Affiliated Hospital of the Zhejiang University School of Medicine (approval number: 529/2022).

### HBV infection

HBV infection of PHHs and NTCP-HepG2 cells was performed as described previously [23]. The HBV virions used for infection were obtained from HepAD38 cells. For HBV infection, PHH cells and NTCP-HepG2 cells were cultured in PMM for 24 h, and then inoculated with HBV particles (MOI = 300 for PHH cells, MOI = 3000 for NTCP-HepG2 cells) in PMM containing 4% PEG8000 at 37 °C for 24 h. After infection, the cells were washed three times with PBS and subsequently cultured in PMM. The culture medium was refreshed every other day.

### Lysosomal fraction extraction

To detect lysosomal proteins, the lysosomal fraction was extracted as previously described [24]. After rinsing with PBS and wash buffer (0.25 M sucrose, 10 mM Tris-HCl), cells cultured in a 10 cm dish were collected using 1 ml homogenization medium (0.25 M sucrose, 1 mM EDTA, 10 mM Tris-HCl, and protease inhibitors, pH 7.4) and homogenized with a Dounce homogenizer. Cell lysates were centrifuged at 2,600 g for 10 min at 4 °C. The supernatant was then centrifuged at 20,000 g for 20 min at 4 °C. The pellet contained lysosomes, mitochondria, endosomes, peroxisomes and other heavy membrane fractions. The remaining supernatant constituted the cytosolic fraction and was further centrifuged at 150,000 g for 1 h at 4 °C to remove light membrane fractions including Golgi apparatus and endoplasmic reticulum.

### Western blotting assays

The methods for preparing whole-cell protein lysates and Western blotting have been described previously [18]. For native PAGE of lysosomal fraction, no denaturing agent or reducing agent was added to the sample loading buffer, PAGE gel and gel running buffer. The information about the antibodies and other reagents is shown in Table S3.

### Detection of HBV gene expression and replication

HBV DNA in the culture supernatant was extracted with a TIANamp Virus DNA/RNA Kit (Tiangen, China) according to the manufacturer’s instructions and quantified by real-time qPCR assay. The HBsAg and HBeAg levels in the culture supernatants were measured by chemiluminescent microparticle immunoassay (CMIA, Abbott Laboratories, USA). To detect HBV DNA replication intermediates (RIs), Southern blotting analysis was performed with a DIG-High Prime DNA Labelling and Detection Starter Kit II (Roche) according to the manufacturer’s instructions, and real-time qPCR was performed using primers specific for HBV DNA. Total RNA was extracted from cells with TRIzol reagent (Invitrogen). HBV RNA levels were measured by real-time reverse transcription (RT)-PCR assays (Takara) using primers specific for HBV RNA (Table S4).

Fluorescence *in situ* hybridization (FISH) was conducted to visualize the intracellular localization of HBV rcDNA using the RNAscope Multiplex Fluorescent Reagent Kit v2 in conjunction with the RNAscope Probe-V-HBV-GTD-sense, following the manufacturer’s instructions. The probe binds to the minus strand of HBV rcDNA (−DNA) in the region directly opposite the single-stranded gap on the plus strand (nt 141–1402), as previously described [25].

### Mitochondrial respiratory complexes I-IV activity assay

The activities of mitochondrial respiratory complexes I-IV were measured using commercial kits (Solarbio) according to a modified method described previously [26]. In brief, ∼1 × 10^7^ cultured cells or ∼100 mg mouse liver tissues were collected with extraction solution in the kits and homogenized using a Dounce homogenizer. Cell lysis was centrifuged at 1,000 g for 10 min at 4 °C. Then, the supernatants were centrifuged at 11,000 g for 20 min at 4 °C to pellet mitochondria. The isolated mitochondria were subjected to three freeze–thaw cycles in liquid nitrogen and a 25 °C water bath to damage the mitochondrial membrane. The activity of mitochondrial respiratory complexes I-IV was then measured according to the manufacturer’s instructions.

### Seahorse mitochondrial stress analysis

The cellular oxygen consumption rate (OCR) was measured using the Seahorse XFe 96 analyzer (Agilent) according to the manufacturer’s instructions. Briefly, NTCP-HepG2 cells were divided into 6 cm plates and infected with HBV virions for 24 h. The medium was changed every other day for 8 days. 2 × 10^4^ cells/well were seeded in the 96-well XF cell culture microplate for 24 h, and the Cell Mito Stress Test Kit was used for the OCR measurement. For PHH, 2 × 10^4^ cells/well were seeded in the 96-well XF cell culture microplate, and infected with HBV for 24 h. The medium was changed every other day for 8 days. Then, the Cell Mito Stress Test Kit was used for the OCR measurement. It measures basal respiration, ATP-linked respiration (inhibited by oligomycin), maximal respiration (stimulated by FCCP). Data analysis was performed with the Seahorse XF Cell Mito Stress Test Report Generator package of Seahorse Wave Desktop software.

### Mitochondria and cellular total ATP detection

Cells were transfected with a mitochondrial-localized ATP probe Mito-AT1.03 and subjected to fluorescence resonance energy transfer (FRET) assay, as previously described, to detect mitochondria-specific ATP production [27]. Mito-AT1.03 plasmid is single fusion protein composed of three functional domains arranged in-frame within one continuous open reading frame (ORF): the N-terminal mseCFPΔC11 (excitation wavelength 435 nm, emission wavelength 475 nm), the ϵ subunit of the FoF1-ATP synthase, and the C-terminal yellow fluorescent protein cp173-mVenus (excitation wavelength 515 nm, emission wavelength 527 nm). When ATP binds, the FoF1-ATP synthase forms a folded structure, bringing the N-terminus CFP and the C-terminus mVenus into close proximity in space, resulting in FRET. Cellular total ATP was detected by an ATP detection Kit (Beyotime, S0026) according to the protocol.

### Immunofluorescence and confocal microscopy

Cells were fixed and permeabilized post transfection or post treatment. After washing and blocking, tissues or cells were incubated with the primary antibodies for 1 h, rinsed with PBS, and incubated with the appropriate AlexaFluor-labeled secondary antibodies for 1 h. Nuclei were stained with DAPI. Following washing and mounting, cells were analyzed under a Leica SP8 DIVE Confocal Microscope or a Zeiss LSM 900 Confocal Microscope. To detect mitochondria-lysosome contacts, cells were cotransfected with LAMP1-mCherry, TOMM20-EGFP plasmid for 2 days. For time-lapse imaging, live cells were cultured in confocal dish and imaged at 5-second intervals 3–5 min under a Zeiss LSM 900 Confocal Microscope with a super-resolution Airyscan-2 module. Contact was defined as the partial overlap between mitochondria and lysosomes. The percentage of lysosomes in contact was quantified as the proportion of lysosomes in contact with mitochondria to all lysosomes at a single time. The duration of contact was quantified as the time difference between the onset of contact and separation. ImageJ software was used to analyze the fluorescence intensity of the target proteins and the colocalization of the target proteins with each other or with organelle marker proteins. The results presented in the graphs were calculated from at least 6 cells.

### Lysosomal pH detection

The LysoSensor Green DND-189 dye was used to quantify the relative pH. It has an in vitro pKa of 5.2 and thus is maximally fluorescent in acidic conditions with absorption and emission peaks of 443 and 505 nm respectively. pH Standards (20 mM 2-(N-Morpholino)ethanesulfonic acid (MES), 110 mM KCl, 20 mM NaCl, 10 μM nigericin and 5 μM monensin, and adjusted to pH 4.0–6.0) were used to plot a standard curve [28] to convert fluorescence intensity to lysosomal pH.

### Transmission electron microscopy imaging

For liver tissue, images were examined under transmission electron microscopy using standard operating procedures [29].

### Cell proliferation assay

Cell proliferation was measured by a Cell Counting Kit-8 Assay (Beyotime, C0038) according to the manufacturer’s protocol.

### Statistical analyses

The results are expressed as the mean ± SEM. Statistical analyses were performed using GraphPad Prism software version 9 (La Jolla, CA, USA). Differences in the means of two groups were determined using the Student’s paired *t* test. Data for single-factor experiments were analyzed using one-way ANOVA. Data for two-factor experiments were analyzed using two-way ANOVA. Differences were considered statistically significant at *p*<0.05. All experiments were independently repeated at least three times.

## Supporting information

Supporting information for this article can be accessed on the publisher's website.

## Results

### Both lysosomes and mitochondria exhibit functional deterioration in HBV-replicating hepatoma cell lines

**Figure 1.**
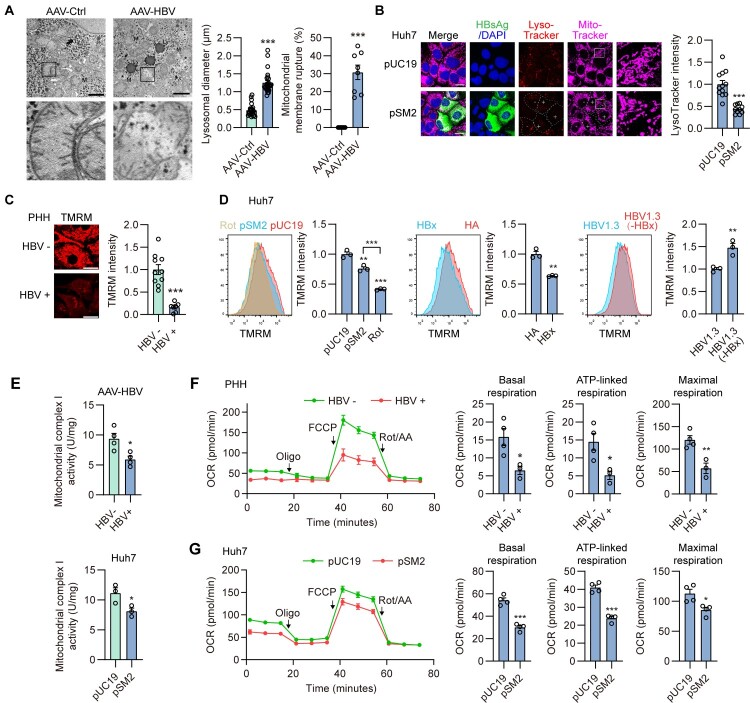
**Both lysosomes and mitochondria exhibit functional deterioration in HBV-replicating hepatoma cell lines.** (A) Representative transmission electron microscopy images of mitochondria (M) and lysosomes (Ly) in AAV-Ctrl and AAV-HBV mouse liver tissues. The black dots are glycogen granules. Black asterisks represented mitochondrial membrane rupture. Scale bar, 500 nm. (B) Huh7 cells were transiently transfected with empty vector (pUC19) or with HBV construct plasmid (pSM2), and collected at 48 h post transfection. The cells were stained with 200 nM MitoTracker for 30 mins and 50 nM LysoTracker Red for 2 h, and then analyzed by confocal microscopy. Scale bar, 20 μm. (C) PHH cells were infected with HBV particles for 4 or 5 days. Mitochondrial membrane potential was measured by staining with TMRM. Scale bar, 20 μm. (D) Huh7 cells were transiently transfected for 2 days under the following experimental setups: (1) control plasmid pUC19 or HBV expression plasmid pSM2; (2) empty vector (HA, control) or HA-tagged HBx expression plasmid (HA-HBx); and (3) HBV expression plasmid HBV 1.3 (wild-type) or HBx-deficient mutant plasmid (HBV1.3 HBx-). TMRM fluorescence was analyzed by flow cytometry to assess mitochondrial membrane potential. Rot was used as positive control. (E) Male C57BL/6 mice (8 weeks old) were transduced with AAV-HBV1.2 through tail vein injection for 4 weeks; Huh7 cells were transiently transfected with pUC19 or with pSM2. Mitochondria were isolated from cell lysis or liver tissues to measure the activity of mitochondrial respiratory chain complexes I. (F) PHH cells were infected with HBV particles, (G) Huh7 cells were transiently transfected with pUC19 or with pSM2. (F, G) The oxygen consumption rate (OCR) was measured by Seahorse Mitochondria Stress Test Kit. *p <0.05; **p <0.01; ***p <0.001; ns, not significant.

Previous studies have shown that HBV infection can lead to lysosomal inactivation and mitochondrial dysfunction [14,18,20,30,31]. However, whether these two processes are functionally connected in the context of HBV infection remains unclear. To address this, we first evaluated lysosomal and mitochondrial function under HBV-infected conditions. In the AAV-HBV mouse model, transmission electron microscopy revealed lysosomal swelling, characterized by a marked increase in lysosomal diameter, indicating lysosomal hypofunction (Figure 1A). Adjacent liver sections were stained using IF for HBsAg (Figure S1A). Consistently, lysosomal activity was reduced in HBV-positive cells, as evidenced by reduced LysoTracker fluorescence intensity, compared to HBV-negative cells (Figure 1B). Consistently, IF staining and Western blotting showed that the levels of both LC3-II and sequestosome-1 (SQSTM1, also known as p62), two typical autophagic proteins that were previously reported to accumulate in damaged lysosomes [32], began to increase immediately after pSM2 transfection in Huh7 cells (Figure S1B). Upon chloroquine (CQ) treatment, both markers further increased, confirming HBV-mediated disruption of autophagic flux (Figure S1C). To identify the specific HBV proteins responsible for the reduction in lysosomal degradation, we transfected Huh7 cells with HBV protein expression plasmids (HA-HBcAg, HA-HBx, HA-Pol, HA-HBeAg, HA-SHBsAg, HA-MHBsAg, and HA-LHBsAg) or an empty vector. Western blotting and IF staining demonstrated that HBx expression dramatically decreased LysoTracker staining and autophagic flux, as indicated by accumulation of both LC3-II and p62 (Figure S1D, S1E). Consistently, HBx impaired autophagic degradation, shown by elevated LC3-II and p62 levels (Figure S1F), hinting that HBx production inhibits autophagic cargo degradation in hepatocytes, consistent with previous studies [14].

In parallel, mitochondrial morphology and function were also evaluated. Transmission electron microscopy revealed that HBV replication caused mild to moderate mitochondrial damage in the AAV-HBV mouse model, as evidenced by partial rupture of the mitochondrial membrane ( Figure 1 A). Similarly, HBV-positive Huh7 cells exhibited pronounced mitochondrial fragmentation ( Figure 1 B). Mitochondrial membrane potential (ΔΨm), which serves as an intermediate energy source for ATP production by mitochondrial ATP synthase, was measured using tetramethyl rhodamine methyl ester (TMRM), a cell-permeant dye that accumulates in active mitochondria. HBV-infected primary human hepatocytes (PHHs) exhibited reduced TMRM fluorescence ( Figure 1 C), indicating a loss of ΔΨm. Elevated levels of HBsAg and HBeAg (Figure S1G) confirmed active viral antigen expression. A similar ΔΨm decline was observed by flow cytometry in transiently HBV-replicating Huh7 cells ( Figure 1 D), supporting HBV-induced mitochondrial dysfunction. Rotenone (Rot), a mitochondrial respiratory complex I inhibitor used as a positive control, induced comparable ΔΨ depolarization. Notably, HBx expression alone significantly reduced TMRM fluorescence, whereas an HBx-lacking HBV mutant restored ΔΨm in Huh7 cells ( Figure 1 D), highlighting the pivotal role of HBx in inducing mitochondrial dysfunction, consistent with its effect on lysosomal dysfunction. To further confirm the mechanism by which HBV replication damages the ΔΨm, the activities of the mitochondrial respiratory complexes I-IV were measured. HBV replication led to a significant reduction in complex I activity in both the AAV-HBV mouse model and HBV-replicating Huh7 cells, whereas the activities of other complexes remained unaffected ( Figure 1 E, S2). Consistently, the Seahorse XF Cellular Mitochondrial Stress assay revealed inhibited basal respiration, ATP-linked respiration, and maximal respiration in HBV-infected PHH and Huh7 cells ( Figure 1 F, G). These findings suggest that HBV replication impairs the mitochondrial respiratory chain and mitochondrial function in hepatocytes.

Taken together, these findings suggest that HBV replication, impairs mitochondrial function and autophagic cargo degradation in hepatocytes.

### Moderate inhibition of mitochondrial respiratory complex I activity enhances HBV replication and secretion by blocking lysosomal activity both *in vitro* and *in vivo*

**Figure 2.**
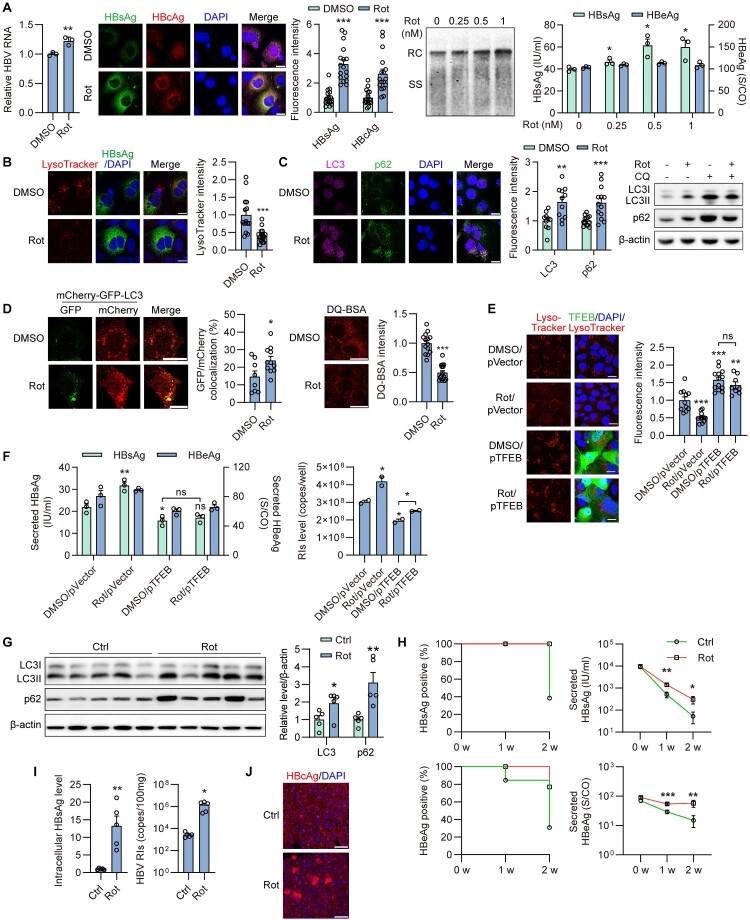
**Moderate inhibition of mitochondrial respiratory complex I activity enhances HBV replication and secretion by blocking lysosomal activity both *in vitro* and *in vivo*.** (A-C) Huh7 cells were transiently transfected with HBV construct plasmid pSM2, then treated with DMSO (ctrl) or Rot (1 nM or indicated concentration) for 2 or 3 days. Scale bar, 20 μm. (A) HBV RNAs quantified by real-time RT-PCR. The HBsAg and HBcAg levels were measured by immunofluorescence. HBV RIs were analyzed by Southern blotting. The HBsAg and HBeAg levels in the culture supernatants were measured by CMIA. (B) The cells were stained with 50 nM LysoTracker Red for 2 h, and then stained with HBsAg antibody. Scale bar, 20 μm. (C) The cells were cotreated with or without CQ. The expression levels of LC3 and p62 were measured by immunofluorescence and Western blotting. (D) The cells were additionally cotransfected with LC3-GFP-mCherry; or the cells were stained with 10 µg/ml DQ-BSA for 1 h. (E, F) Huh7 cells were transiently cotransfected with pSM2 and empty vector or TFEB plasmid (pTFEB), then treated with DMSO or Rot. (E) The cells were stained with 50 nM LysoTracker Red. Scale bar, 20 μm. (F) The HBsAg and HBeAg levels in the culture supernatants were measured as described above. HBV RIs were extracted and quantified by qPCR. (G-J) C57BL/6 mice received hydrodynamic injection (HDI) with 10 μg of pAAV-HBV1.2 plasmid. 1 week after HDI, the mice were treated with CMC-Na (Ctrl, n = 13) or 8 mg/kg Rot (n = 13) by daily oral gavage for 2 weeks. (G) The expression levels of LC3 and p62 were measured by Western blotting. (H, I) The HBsAg and HBeAg positive rate was calculated at 0, 1 and 2 weeks after CMC-Na or Rot treatment. The levels of HBsAg and HBeAg in the serum and liver lysates were assayed. The levels of HBV RIs were analyzed by qPCR. (J) Intracellular HBcAg was detected by immunofluorescence. Scale bar, 100 μm. *p <0.05; **p <0.01; ***p <0.001; ns, not significant.

To investigate the impact of mitochondrial damage on HBV replication, Rot, a popular inhibitor of respiratory complex I, was used to mimic the mitochondrial dysfunction associated with HBV replication. We first confirmed the efficacy and safety of Rot treatment in Huh7 cells. Rot effectively inhibited mitochondrial complex I activity at low concentrations (Figure S3A), without affecting Huh7 cell viability at doses up to 2.5 nM (Figure S3B). Given that chronic HBV infection does not cause overt hepatocyte damage, a moderate concentration of Rot (≤1 nM) was selected for subsequent experiments in Huh7 cells to induce mild or moderate mitochondrial dysfunction while preserving cell viability. Rot treatment increased intracellular HBsAg and HBcAg levels, intracellular HBV RIs, and secreted HBsAg levels (Figure 2A, S3C), while moderately increasing total HBV RNA levels and not affecting secreted HBeAg. This is consistent with our previous findings that autophagy has minimal impact on HBV transcription and HBeAg secretion. Furthermore, Rot treatment enhanced HBV production in the HBV-infected NTCP-HepG2 cells (Figure S3D).

Mitochondria and lysosomes undergo concurrent inactivation in HBV-infected cells. Previously, we demonstrated that blocking autophagic degradation enhanced HBV replication and secretion [14,17,19,23]. Thus, we explored whether Rot enhanced HBV replication and progeny release via inhibiting lysosomal degradation in hepatoma cells. Firstly, lysosomal activity was measured after Rot treatment. LysoTracker fluorescence intensity was decreased following Rot treatment (Figure 2B). Additionally, IF staining and Western blotting demonstrated elevated LC3 and p62 levels after Rot treatment (Figure 2C). Upon subsequent CQ cotreatment, both markers further increased, indicating a blockade of autophagic flux and impaired degradation of autophagic cargo. To further confirm the inactivation of lysosomes, mCherry-GFP-LC3 plasmid and dye-quenched bovine serum albumin (DQ-BSA) were used to measure the lysosomal degradation capacity. Rot induced the accumulation of autophagosomes, with enhanced expression of both mCherry and GFP (Figure 2D). Furthermore, the fluorescence signal of DQ-BSA produced by autolysosomal proteolysis was decreased after Rot treatment (Figure 2D), indicating incomplete autophagy flux and reduced cargo degradation in autophagosomes. Next, to demonstrate that Rot-promoted HBV replication is through the regulation of autophagic flux, we overexpressed transcription factor EB (TFEB, a master regulator of lysosome biogenesis) to restore Rot-inhibited autophagic flux. Overexpression of TFEB in HBV-replicating Huh7 cells restored the Rot-decreased lysosomal activity (Figure 2E). Additionally, TFEB expression blocked the Rot-induced elevation of secreted HBsAg and intracellular HBV RIs levels (Figure 2F), indicating that the mitochondria control HBV replication by regulating autophagic degradation.

Subsequently, we further confirmed these effects in an HBV hydrodynamic injection (HDI) mouse model. No significant changes in body weight or in serum ALT and AST levels were observed after Rot treatment compared to the control group, indicating that Rot did not cause apparent toxicity in mice (Figure S3E, S3F). Additionally, Rot treatment increased LC3-II and p62 levels ( Figure 2 G), further supporting lysosomal inactivation after Rot treatment *in vivo*. Furthermore, the HBV-positive rate exhibited a significant increase compared to the untreated group during the 2-week observation period following Rot treatment ( Figure 2 H). It is worth noting that secreted HBsAg, HBeAg, and intracellular HBsAg exhibited a significant increase during this period ( Figure 2 H, 2I). Additionally, intracellular HBV RIs showed substantial elevation after Rot treatment ( Figure 2 I). In mouse liver tissues, HBcAg primarily accumulated in the nucleus in the control group. Whereas in the Rot-treated group, a large amount of HBcAg was expressed and extended to the cytoplasm ( Figure 2 J), indicating more active viral replication.

Taken together, our data demonstrate that blocking mitochondrial function with Rot treatment promotes HBV production via blocking lysosomal activity both *in vitro* and *in vivo*.

### Blocking mitochondrial reactive oxygen species (mtROS) clearance inhibits lysosomal activity and enhances HBV replication in hepatoma cell lines

**Figure 3.**
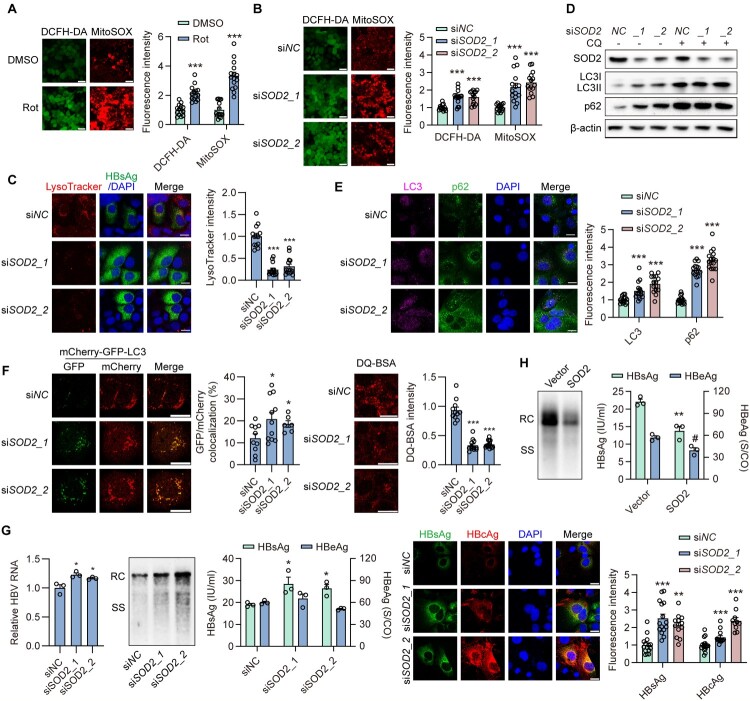
**Blocking mitochondrial reactive oxygen species (mtROS) clearance inhibits lysosomal activity and enhances HBV replication in hepatoma cell lines.** (A) Huh7 cells were transiently transfected with pSM2, then treated with or without Rot for 2 days. DCFH-DA and MitoSOX staining were used to detect total ROS levels and mtROS levels, respectively. Scale bar, 50 μm. (B-G) Huh7 cells were transiently cotransfected with 40 nM siSOD2_1, siSOD2_2 or negative control siRNA (siNC), and HBV construct plasmid pSM2 for 2 or 3 days. (B) DCFH-DA and MitoSOX staining were used to detect total ROS levels and mtROS levels, respectively. Scale bar, 50 μm. (C) The cells were stained with 50 nM LysoTracker Red for 2 h. Scale bar, 20 μm. (D) The cells were cotreated with or without CQ. The expression of indicated proteins was measured by Western blotting. (E) The fluorescence intensity of LC3 and p62 was analyzed by immunofluorescence. Scale bar, 20 μm. (F) The cells were additionally cotransfected with LC3-GFP-mCherry or stained with 10 µg/ml DQ-BSA for 1 h. (G) HBV RNAs were extracted and quantified by real-time RT-PCR. HBV RIs were analyzed by Southern blotting. The HBsAg and HBeAg levels in the culture supernatants were measured. Intracellular HBsAg and HBcAg were imaged by immunofluorescence. Scale bar, 20 μm. (H) Huh7 cells were transiently cotransfected with SOD2 overexpression plasmid and HBV construct plasmid (pSM2) for 3 days. The levels of HBV RIs and secreted HBsAg and HBeAg were measured. *, #*p* <0.05; **p <0.01; ***p <0.001; ns, not significant.

Dysfunctional mitochondria produce excessive amounts of ROS [33]. Indeed, experiments using 2’, 7’-dichlorofluorescin diacetate (DCFH-DA), a cell-permeable probe of intracellular ROS, and MitoSOX Red dye, a probe of mitochondrial superoxide production in live cells, showed that intracellular and mitochondrial ROS levels were elevated in transiently HBV-replicating Huh7 cells or Rot-treated cells compared with those in control cells (Figure S4A, Figure 3A). This indicates that blocking mitochondrial respiratory complex I by HBV replication or Rot treatment induces increased total and mitochondrial ROS levels. Therefore, to investigate the mechanism by which mitochondrial dysfunction regulates lysosomal activity, the impact of mitochondrial ROS (mtROS) on lysosomal function was explored.

Superoxide dismutase 2 (SOD2) is the primary antioxidant enzyme in alleviating oxidative stress by converting mtROS into hydrogen peroxide and diatomic oxygen. Therefore, we investigated the effects of mtROS on lysosomal activity after SOD2 knockdown. siSOD2_1 and siSOD2_2 were used to knockdown SOD2 expression. Western blotting analysis indicated that both siSOD2_1 and siSOD2_2 effectively decreased SOD2 expression (Figure S4B). DCFH-DA and MitoSOX staining revealed that SOD2 knockdown significantly elevated both total ROS and mtROS levels ( Figure 3 B). Next, we assessed lysosomal activity following SOD2 knockdown. IF staining demonstrated reduced LysoTracker fluorescence intensity upon SOD2 knockdown ( Figure 3 C), indicating compromised lysosomal function. In parallel, both LC3 and p62 levels were significantly increased in SOD2-knockdown Huh7 cells, as evidenced by Western blotting and IF staining ( Figure 3 D, E). Consistently, SOD2 knockdown induced the accumulation of autophagosomes, evidenced by increased GFP^+^ mCherry^+^ LC3 puncta, and decreased the fluorescent signal of DQ-BSA ( Figure 3 F), indicating defective cargo degradation and incomplete autophagic processing. These results indicate that SOD2 knockdown results in the co-dysfunction of mitochondria and lysosomes.

Next, we explored whether silencing SOD2, which inhibits lysosomal activity, leads to elevated HBV replication and progeny release in hepatoma cells. Intracellular HBV RIs, and secreted HBsAg levels, along with intracellular HBsAg and HBcAg levels, were significantly increased in SOD2-knockdown Huh7 cells ( Figure 3 G, S4C), while causing a moderate increase in total HBV RNA levels without affecting the secretion of HBeAg. Similarly, SOD2 knockdown enhanced HBV production in the HBV-infected NTCP-HepG2 cells (Figure S4D). Conversely, SOD2 overexpression reduced HBV replication, as evidenced by the decreased HBV RIs and secreted HBsAg and HBeAg levels in SOD2-overexpressing Huh7 cells ( Figure 3 H).

### mtROS-mediated lysosomal membrane permeabilization plays a key role in rot-inhibited lysosomal degradation and enhanced HBV replication

The normal capacity for lysosomal degradation plays a crucial role in the terminal compartment during autophagy. Studies have demonstrated that lysosomal membrane permeabilization (LMP) and acidic pH loss within lysosomes impair the lysosomal degradation capacity [34]. In addition, it has been reported that ROS can trigger LMP [35,36]. To confirm whether HBV regulates lysosomal stability, we measured lysosomal pH and membrane integrity by observing the fluorescence of LysoSensor Green DND-189 (pH indicator) and the condensation of Galectin-3-EGFP (a cytoplasmic lectin that binds to lysosomal polysaccharides and becomes exposed once the lysosomal membrane is damaged). Acidification of the lumen of lysosomes to a pH of 4.5–5.0 is essential for their function [37]. The lysosomal pH value of Huh7 cells transfected with an empty vector remained at the normal value (around 4.60), while HBV-replicating Huh7 cells showed an increase of lysosomal pH value around 5.65 (Figure S5A). Additionally, the average number of Galectin-3 puncta and the colocalization ratio of Galectin-3 and LAMP1 were obviously increased in HBV-replicating Huh7 cells, confirming a large amount of lysosomal membrane damage due to HBV replication (Figure S5B). L-Leucyl-L-leucine methyl ester (LLOMe), the well-characterized lysosomotropic agent that polymerizes inside lysosomes to induce fast but reversible lysosomal membrane damage, was used as a positive control.

**Figure 4.**
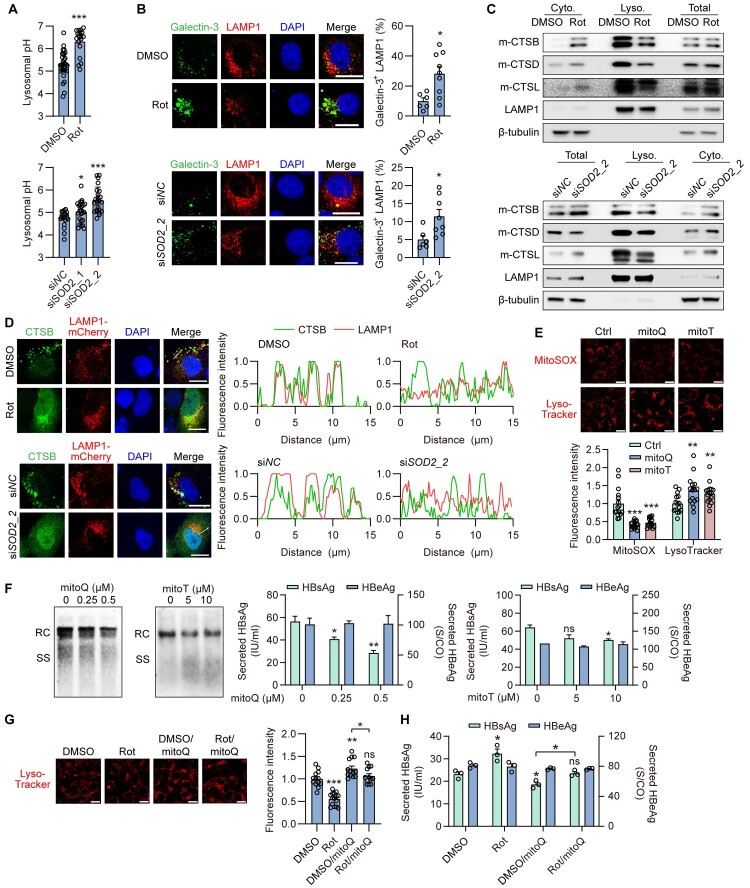
**mtROS-mediated lysosomal membrane permeabilization plays a key role in HBV-inhibited lysosomal degradation.** (A-D) Huh7 cells were transiently transfected with pSM2, then treated with Rot or cotransfected with 40 nM siSOD2_1, siSOD2_2. (A) The cells were stained with 1 µM LysoSensor Green DND-189 for 1 h and analyzed by confocal microscopy. The fluorescence intensity was converted into lysosomal pH according to the standard curve. (B) The cells were additionally cotransfected with Galectin-3-EGFP plasmid. Colocalization of Galectin-3 and LAMP1 was analyzed. (C) Mature CTSD (m-CTSD) and mature CTSL (m-CTSL) in the cytosol (Cyto.), lysosomal (Lyco.) fraction and total cell lysates were measured by Western blotting. (D) The cells were co-transfected with LAMP-mCherry plasmid. The colocalization of CTSB and LAMP1 was measured by immunofluorescence. (E-F) Huh7 cells were transiently transfected with HBV construct plasmid pSM2, then treated with indicated concentration of mitoQ or mitoT for 2 or 3 days. (E) The cells were stained with MitoSOX for 30 mins, or 50 nM LysoTracker Red for 2 h, respectively. (F) The levels of HBV RIs were analyzed by Southern blotting. The secreted HBsAg and HBeAg were measured. (G, H) Huh7 cells were transiently transfected with HBV construct plasmid pSM2, then cotreated with indicated concentration of mitoQ and Rot. (G) The cells were stained with 50 nM LysoTracker Red for 2 h. (H) The secreted HBsAg and HBeAg levels were measured. Scale bar, 20 μm. *p <0.05; **p <0.01; ***p <0.001; ns, not significant.

Next, we investigated the effect of Rot treatment or SOD2 knockdown, which blocks mitochondrial function by increasing both total and mitochondrial ROS levels, on lysosomal stability. LysoSensor Green DND-189 staining revealed that lysosomal pH value in Huh7 cells treated with Rot or SOD2 knockdown was further increased compared to cells transfected with HBV alone (Figure 4A). Similarly, the average number of Galectin-3 puncta and the colocalization ratio of Galectin-3 and LAMP1 were increased in Rot-treated or SOD2 knockdown Huh7 cells (Figure 4B), demonstrating that Rot treatment or SOD2 knockdown induced LMP. To further confirm LMP after Rot treatment or SOD2 knockdown, we isolated the lysosomal fraction and detected the lysosomal hydrolase levels. Levels of mature-CTSB (m-CTSB, cathepsin B), mature-CTSD (m-CTSD, cathepsin D) and mature-CTSL (m-CTSL, cathepsin L), three major lysosomal proteases, were increased in the cytoplasm and decreased in the lysosomal fraction after Rot treatment or SOD2 knockdown (Figure 4C). Additionally, Rot treatment or SOD2 knockdown disrupted the co-localization of CTSB and LAMP1, as shown by reduced overlap in fluorescence signals (Figure 4D). These data suggest that lysosomal contents leak into the cytoplasm in Rot-treated or SOD2-knockdown Huh7 cells.

Subsequently, we investigated whether Rot treatment inhibits lysosomal activity and promotes viral replication by inducing mtROS and LMP in host cells. The mitochondria-targeted antioxidants mitoquinone (mitoQ) and mitoTEMPO (mitoT) were used to scavenge mtROS. Indeed, the fluorescence intensity of MitoSOX was decreased upon addition of mitoQ or mitoT to cultured hepatoma cells ( Figure 4 E), indicating effective scavenging of mtROS. To assess the impact on LMP, Galectin-3 puncta formation and its colocalization with LAMP1 were measured. Both the average number of Galectin-3 puncta and the colocalization ratio between Galectin-3 and LAMP1 were decreased following mitoQ or mitoT treatment (Figure S6A), suggesting alleviation of LMP. Consistently, LysoTracker fluorescence intensity was increased in HBV-transfected Huh7 cells treated with mitoQ or mitoT ( Figure 4 E), indicating restored lysosomal function. Moreover, LC3-II and p62 expression levels were markedly decreased after mitoQ or mitoT treatment, but significantly upregulated upon co-treatment with CQ, (Figure S6B), confirming that scavenging mtROS restored lysosomal activity. Importantly, mitoQ or mitoT treatment also reduced the levels of intracellular HBV RIs and secreted HBsAg ( Figure 4 F). In HBV-transfected Huh7 cells cotreated with Rot, mitoQ partially reversed Rot-induced lysosomal dysfunction, as evidenced by LysoTracker fluorescence intensity remaining nearly unchanged compared to untreated cells ( Figure 4 G). Furthermore, mitoQ partially abolished Rot-induced upregulation of HBsAg secretion ( Figure 4 H).

Taken together, these results suggest that mtROS-mediated LMP play a key role in Rot-inhibited lysosomal degradation in hepatoma cell lines.

### Disruption of mitochondrial ATP synthesis leads to lysosomal inactivation and enhances HBV replication in an mtROS-independent manner

**Figure 5.**
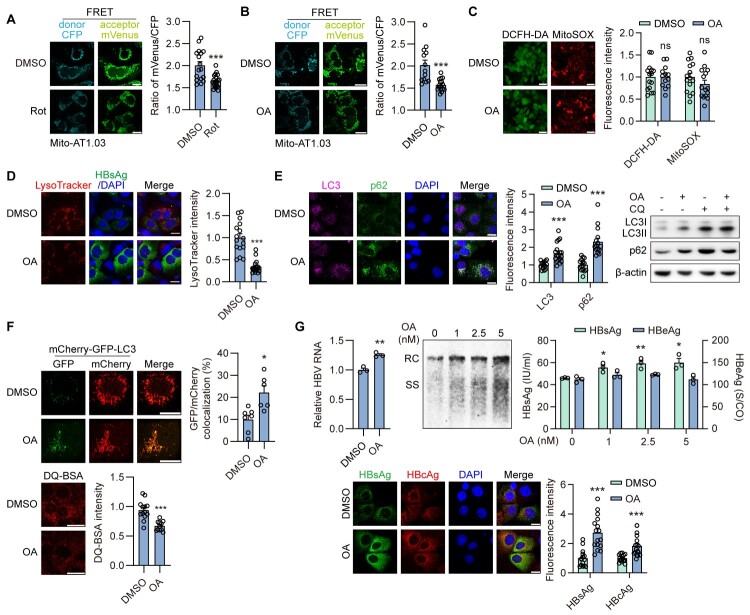
**Disruption of mitochondrial ATP synthesis leads to lysosomal inactivation and enhances HBV replication in an mtROS-independent manner.** (A, B) Huh7 cells were transfected with Mito-AT1.03 plasmid, and then treated with Rot (1 nM) or OA (5 nM) for 2 days. Mitochondria-specific ATP production was detected as described. Scale bar, 20 μm. (C-G) Huh7 cells were transiently transfected with pSM2, then treated with OA (5 nM) for 2 or 3 days. (C) DCFH-DA and MitoSOX staining were used to detect total ROS levels and mtROS levels, respectively. Scale bar, 50 μm. (D) The fluorescence intensity of LysoTracker was detected as described above. Scale bar, 20 μm. (E) The cells were cotreated with or without CQ. Immunofluorescence and Western blotting were used to measure the indicated proteins. Scale bar, 20 μm. (F) The cells were additionally cotransfected with LC3-GFP-mCherry (upper). The cells were stained with 10 µg/ml DQ-BSA for 1 h (lower). Scale bar, 20 μm. (G) HBV RNAs were extracted and quantified by real-time RT-PCR. HBV RIs were analyzed by Southern blotting. Intracellular HBsAg and HBcAg were imaged by immunofluorescence. The HBsAg and HBeAg levels in the culture supernatants were measured. Scale bar, 20 μm. *p <0.05; **p <0.01; ***p <0.001; ns, not significant.

Mitochondria are the primary sites of ATP production in mammalian cells. In addition to mtROS, mitochondrial ATP production is also inhibited by HBV replication or respiratory complex I inhibition (Figure S7A, Figure 5A). The role of mitochondrial ATP in regulating lysosomal activity remains unclear. Consequently, we investigated whether mitochondrial ATP influences lysosomal function. Oligomycin A (OA), an inhibitor of mitochondrial ATP synthase, disrupts oxidative phosphorylation and ATP-dependent processes occurring at the mitochondrial membrane. The viability of Huh7 cells was measured after OA treatment at concentrations ranging from 1 nM to 20 nM. In Huh7 cells, OA treatment at concentrations of up to 5 nM did not affect cell viability (Figure S7B). Notably, OA treatment reduced the mVenus/CFP emission ratio (Figure 5B), indicating impaired ATP production in the mitochondria. Surprisingly, DCFH-DA and MitoSOX staining results revealed that OA treatment did not alter either total or mitochondrial ROS levels (Figure 5C). Subsequently, the effects of OA treatment on lysosomal activity were assessed. IF staining demonstrated that LysoTracker fluorescence intensity was reduced after OA treatment (Figure 5D). Additionally, LC3 and p62 levels were markedly increased, as evidenced by IF staining and Western blotting in OA-treated Huh7 cells (Figure 5E). Consistently, OA treatment induced the accumulation of LC3 puncta with enhanced expression of both mCherry and GFP, and decreased the fluorescent signal of DQ-BSA (Figure 5F), indicating incomplete autophagy and reduced cargo degradation in autophagosomes of OA-treated Huh7 cells. These findings highlight the crucial role of mitochondrial ATP in maintaining lysosomal function without altering mtROS levels.

Next, we explored whether mitochondrial ATP influences HBV replication and progeny release in hepatoma cells. OA treatment increased intracellular HBsAg and HBcAg levels, intracellular HBV RIs, and secreted HBsAg levels ( Figure 5 G, Figure S7C), while slightly enhancing total HBV RNA levels and not affecting secreted HBeAg. Moreover, OA treatment enhanced HBV production in the HBV-infected NTCP-HepG2 cells (Figure S7D).

### Mitochondria-lysosome contacts are involved in regulation of lysosomal activity and HBV autophagic degradation

**Figure 6.**
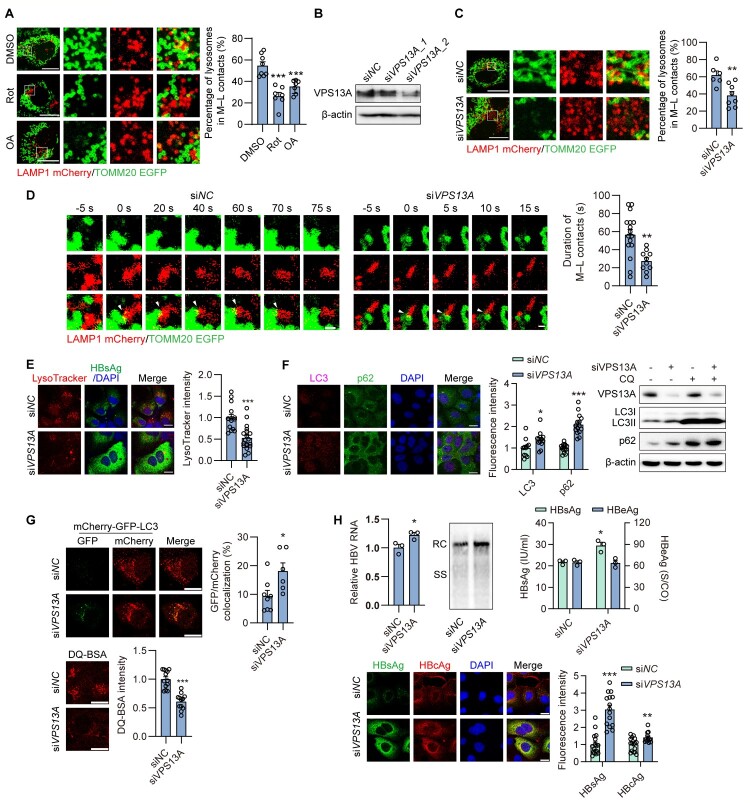
**Mitochondria-lysosome contacts are involved in regulation of lysosomal activity and HBV autophagic degradation.** (A) Huh7 cells were cotransfected with LAMP1-mCherry and TOMM20-EGFP plasmid, subsequently treated with Rot or OA for 2 days. Mitochondria-lysosome contacts were quantified by confocal time lapse images. (B-H) Huh7 cells were transiently cotransfected with 40 nM siVPS13A or siNC and HBV construct plasmid pSM2 for 2 or 3 days. (B) Western blotting was used to measure the indicated proteins. (C, D) Representative time lapse images of a living Huh7 cell expressing TOMM20-EGFP, LAMP1-mCherry after VPS13A knockdown were captured. (C) The percentage of lysosomes in mitochondria-lysosome contacts was analyzed. (D) Quantitation of duration of mitochondria-lysosome contacts within the cytosol from time lapse images. (E) The fluorescence intensity of LysoTracker was detected as described above. (F) The cells were cotreated with or without CQ. The distribution of LC3 and p62 was measured by immunofluorescence microscopy and Western blotting. (G) The cells were additionally cotransfected with LC3-GFP-mCherry (upper). The cells were stained with 10 µg/ml DQ-BSA for 1 h (lower). (H) HBV RNAs were extracted and quantified by real-time RT-PCR. HBV RIs were analyzed by Southern blotting. The HBsAg and HBeAg levels in the culture supernatants were measured. Intracellular HBsAg and HBcAg were imaged by immunofluorescence. Scale bar, 20 μm. *p <0.05; **p <0.01; ***p <0.001; ns, not significant.

Although OA decreased mitochondrial ATP levels, it increased total intracellular ATP levels (Figure S8A). Additionally, the total ATP levels remained unchanged after Rot treatment and HBV gene expression (Figure S8A, S8B). These results implied an increase in ATP production from glycolysis after mitochondrial disruption. However, ATP production from glycolysis cannot rescue impaired lysosomal activity, indicating that lysosomes specifically require mitochondrial ATP production. However, the process by which mitochondrial ATP is transported to lysosomes is not fully understood. It has been reported that mitochondria and lysosomes physically contact each other [10], and that their membrane contacts may affect lysosomal activity [11,12]. Thus, we speculated that mitochondria-lysosome physical contacts may facilitate the flow of mitochondrial ATP to lysosomes (Figure S9A). Indeed, we found that most active lysosomes (indicated by LysoSensor) were predominantly located near mitochondria (Figure S9B). Additionally, HBV replication revealed reduced mitochondrial-lysosomal contacts (Figure S9C). Furthermore, live-cell imaging revealed reduced proportion and duration of LAMP1-positive vesicles in contact with mitochondria in Rot or OA treated Huh7 cell (Figure 6A, S9D). Therefore, we investigated whether mitochondria-lysosome contacts play a role in lysosomal activity and HBV autophagic degradation.

Vacuolar protein sorting 13 A (VPS13A), located in the outer mitochondrial membrane, plays a key role in these contacts [38,39]. siVPS13A_1 and siVPS13A_2 were used to knockdown VPS13A expression. Western blotting analysis indicated that only siVPS13A_2 effectively decreased VPS13A expression levels (Figure 6B). Thus, siVPS13A_2 was selected for the VPS13A knockdown experiments. Live-cell imaging revealed reduced LAMP1-positive vesicles in contact with mitochondria in VPS13A knockdown Huh7 cells, along with shorter contact duration in the cytoplasm (Figure 6C, D), indicating reduced contacts between mitochondria and lysosomes. Notably, similar to OA treatment, VPS13A knockdown did not change either total or mitochondrial ROS levels, as DCFH-DA and MitoSOX staining fluorescence intensity remained unchanged (Figure S10A). Subsequently, the effect of the VPS13A knockdown on lysosomal activity was measured. IF staining showed that LysoTracker fluorescence intensity was decreased after VPS13A knockdown (Figure 6E). Furthermore, p62 levels were markedly increased, as evidenced by Western blotting and IF staining in VPS13A knockdown Huh7 cells (Figure 6F). Consistently, VPS13A knockdown induced the accumulation of LC3 puncta with enhanced expression of both mCherry and GFP, and decreased the fluorescent signal of DQ-BSA (Figure 6G), indicating incomplete autophagy and reduced cargo degradation in autophagosomes. These results indicate that mitochondrial – lysosome contacts play crucial roles in maintaining lysosomal activity.

Subsequently, the effect of VPS13A knockdown on HBV replication and progeny release in hepatoma cells was investigated. Specifically, VPS13A knockdown resulted in elevated intracellular HBV RIs, increased intracellular levels of HBsAg and HBcAg, and increased secretion of HBsAg ( Figure 6 H, S10B) in HBV-replicating Huh7 cells, while leading to a moderate increase in total HBV RNA levels and having no impact on secreted HBeAg. Moreover, our study on HBV-infected NTCP-HepG2 cells demonstrated that VPS13A knockdown significantly increased HBV components compared with controls (Figure S10C).

### Disrupting mitochondrial function elevates lysosomal pH by regulating vacuolar (H+)-adenosine triphosphatase (v-ATPase) assembly

**Figure 7.**
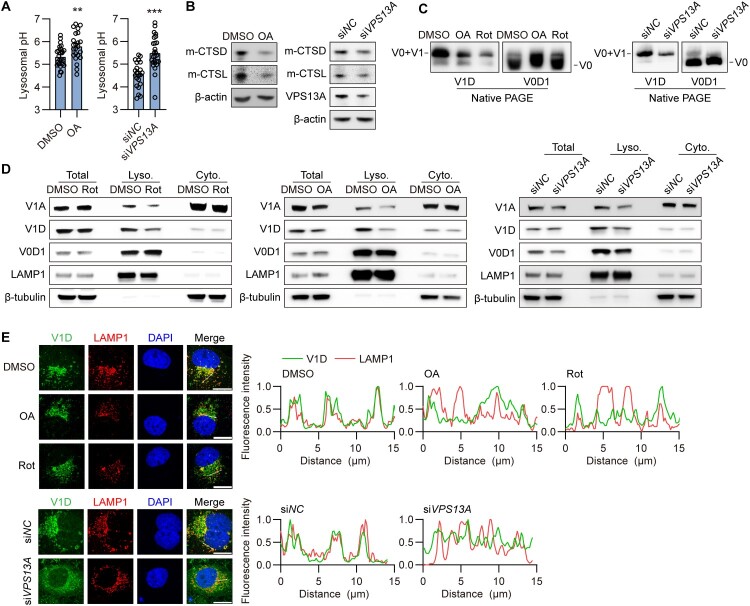
**Disrupting mitochondrial function elevates lysosomal pH by regulating v-ATPase assembly.** (A-F) Huh7 cells were transiently cotransfected with HBV construct plasmid (pSM2), then treated with OA or Rot, or additionally cotransfected with 40 nM siVPS13A. (A) The pH levels were detected as described above. (B) Western blotting was used to measure the indicated proteins. (C) Lysosomes were isolated, and the lysosomal fraction was subjected to native PAGE, and ATP6V1D (V1D) and ATP6V0D1 (V0D1) were used to indicate V-ATPase V1 subunit and V0 subunit, respectively. (D) Lysosomes were isolated, and the levels of ATP6V1A (V1A), V1D and V0D1 in the cytosol (Cyto.), lysosomal (Lyco.) fraction and total cell lysates were measured by Western blotting. (E) The distribution of V1D and LAMP1 was imaged by immunofluorescence microscopy. Scale bar, 20 μm. *p <0.05; **p <0.01; ***p <0.001; ns, not significant.

As shown in Figures 5 and 6, blocking mitochondrial ATP synthesis with OA treatment or disrupting mitochondria-lysosome contacts by VPS13A knockdown did not alter mtROS levels. Additionally, the lysosomal membrane remained impermeable following OA treatment or VPS13A knockdown, as indicated by the unchanged average number of Galectin3-EGFP puncta (Figure S11). However, the lysosomal pH was elevated in Huh7 cells subjected to either OA treatment or VPS13A knockdown, indicating decreased lysosomal activity (Figure 7A). Additionally, OA treatment and VPS13A knockdown significantly inhibited the cleavage of CTSD and CTSL (Figure 7B). These data suggest that other mechanisms may be involved in the regulation of lysosomal acidification.

Lysosomal acidification primarily relies on vacuolar (H+)-adenosine triphosphatase (v-ATPase), a multimeric enzyme complex responsible for pumping protons from the cytosol into the lysosomal lumen [37]. v-ATPases are ATP-driven proton pumps composed of an integral membrane-associated V0 domain and a cytoplasmic V1 domain. Its activity relies on the assembly of the V0 domain with the V1 domain at the lysosomal membrane [40,41]. To explore v-ATPase assembly, native polyacrylamide gel electrophoresis (PAGE) was performed. On native PAGE gels, the fully assembled v-ATPase was significantly reduced in Rot- or OA-treated cells compared to control cells (Figure 7C). In contrast, the V0 domain associated with lysosomal membranes was not affected. Consistently, VPS13A knockdown yielded similar findings (Figure 7C). To further investigate the possible disruption of v-ATPase assembly after Rot or OA treatment or VPS13A knockdown, we examined the assembly of the v-ATPase complex on isolated lysosomal membranes. Western blotting analysis revealed significantly lower levels of V1A and V1D on lysosomal membranes in cells treated with Rot, OA, or subjected to VPS13A knockdown (Figure 7D), whereas V0D1 remained unchanged. Consistently, IF staining demonstrated that the colocalization of V1D and LAMP1 was markedly decreased in these cells (Figure 7E), suggesting that the V1 domain failed to assemble with the membrane-bound V0 domain.

In summary, these data demonstrate that disrupting mitochondrial function or its contact with lysosomes leads to an increase in lysosomal pH by modulating the v-ATPase assembly.

### Moderate inhibition of mitochondrial complex I enhances endosomal trafficking of HBV components

**Figure 8.**
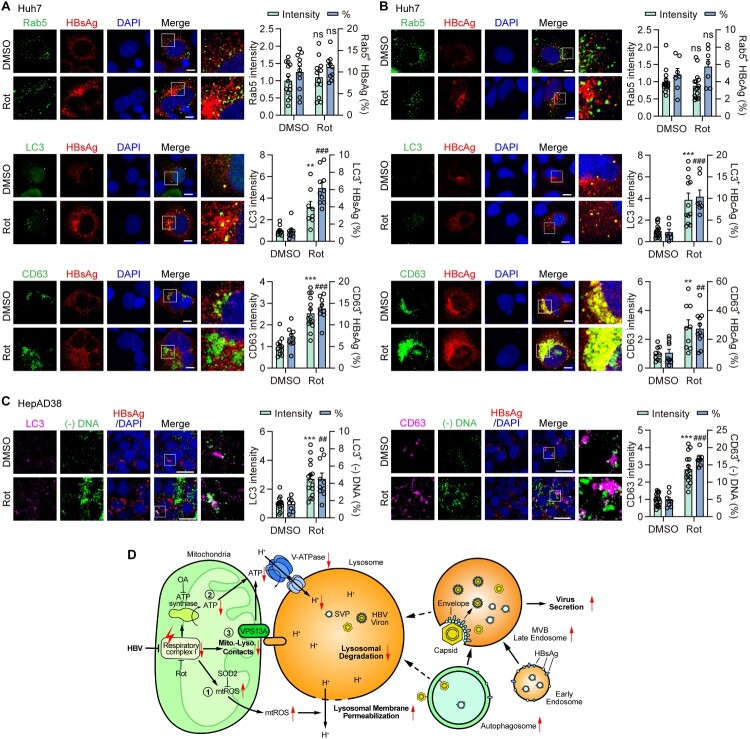
**Moderate inhibition of mitochondrial complex I enhances endosomal trafficking of HBV components.** (A, B) Huh7 cells were transiently cotransfected with HBV construct plasmid pSM2 and Rab5A-EGFP (early endosomes), LC3-EGFP (autophagosomes) or CD63-EGFP (MVBs), then treated with DMSO or Rot (1 nM) for 2 days. The effects of Rot on Rab5A puncta intensity (upper), LC3 puncta intensity (middle), and CD63 puncta intensity (lower) and their colocalization with HBsAg (A) or HBcAg (B) were evaluated. (C) HepAD38 cells were treated with Rot for 2 days. Cells were subjected to RNAscope fluorescence *in situ* hybridization (FISH) using a probe targeting the single-stranded region of HBV rcDNA. Then the cells were stained with the indicated proteins. Fluorescence signals were visualized by confocal microscopy. Scale bar, 10 μm. (D) Schematic diagram of mitochondrial respiratory chain regulation of HBV clearance through lysosomal pathways. *,#*p* <0.05; **,##*p* <0.01; ***,###*p* <0.001.

Autophagosomes are capable of transporting HBsAg and naked capsids, and subsequently fuse with multivesicular bodies (MVBs), a process that becomes particularly evident when lysosomal function is inhibited, thereby contributing to the secretion of HBV components [14]. Previous studies have reported that MVBs serve as the primary platform for capsid envelopment and mature virion release [14,42]. Thus, we speculated that the promotion of HBV replication and secretion upon inhibition of mitochondrial respiratory complex I might be associated with changes in the vesicular trafficking of HBV components. To test this hypothesis, we treated HBV-replicating Huh7 cells with Rot and examined the colocalization of HBsAg with markers of early endosomes (Rab5A), autophagosomes (LC3), and MVBs (CD63). Rot treatment did not alter the fluorescence intensity of Rab5A or its colocalization with HBsAg (Figure 8A, upper). In contrast, HBsAg colocalization with LC3 increased ∼3.3-fold, accompanied by elevated LC3 puncta intensity, despite a limited proportion of colocalization (Figure 8A, middle). Similarly, HBsAg colocalization with CD63 approximately doubled, along with enhanced CD63 puncta fluorescence (Figure 8A, lower). HBcAg exhibited similar patterns: its colocalization with Rab5A remained unchanged, with a very low colocalization ratio (Figure 8B, upper), while colocalization with LC3 and CD63 significantly increased following Rot treatment (Figure 8B, middle and lower), indicating enhanced recruitment into autophagosomes and MVBs. Notably, the colocalization ratio of HBcAg with CD63 increased from 10.6% to 27.4% in Rot-treated Huh7 cells (Figure 8B), suggesting that inhibition of mitochondrial respiratory complex I facilitates capsid entry into the endosomal system. These findings collectively indicate that Rot treatment promotes the accumulation of vesicular compartments and enhances the incorporation of HBV components into autophagosomes and MVBs.

To unequivocally demonstrate the association of HBV genomic material with the autophagy – MVB pathway, we examined the spatial colocalization of HBsAg, and HBV DNA with MVBs and autophagosomes. HBV DNA was visualized using RNAscope *in situ* hybridization. Probe specificity was validated using HepAD38 cells under Tet-regulated HBV replication: rcDNA signals were absent in Tet (+) cells but clearly detected in Tet (–) cells, confirming active replication and reliable detection method (Figure S12). To minimize potential artifacts from pSM2 plasmid transfection in Huh7 cells, particularly affecting HBV DNA quantification, we utilized the HepAD38 cell line, which supports inducible HBV replication [43]. Notably, the results revealed that Rot treatment markedly enhanced the colocalization of HBV DNA with LC3 and CD63, accompanied by increased fluorescence intensity and colocalization ratios (Figure 8C). These findings support the notion that HBV genomic material is trafficked through autophagosomes and MVBs, potentially facilitating viral processing or egress.

Collectively, these findings suggest that disruption of the mitochondrial respiratory chain facilitates the trafficking of HBV particles into the endosomal pathway, thereby enhancing their secretion.

## Discussion

In the present study, we demonstrated that HBV replication inhibited mitochondrial complex I activity and mitochondrial function both *in vitro* and *in vivo*, resulting in increased mtROS, reduced mitochondrial ATP production, and decreased mitochondria-lysosome contacts, thereby impairing HBV autophagic degradation in hepatoma cell lines. Elevated mtROS levels resulted in deteriorated LMP and increased HBV production. Additionally, blocking mitochondrial ATP production and mitochondria-lysosome contacts reduced the lysosomal degradation capacity by inhibiting v-ATPase assembly. Ultimately, inhibition of mitochondrial complex I facilitated HBV secretion by promoting the endosomal trafficking of HBV components ( Figure 8 D). These findings provide a plausible explanation for the coordinated control of HBV autophagic degradation by mitochondria and lysosomes through regulation of LMP and v-ATPase assembly.

Mitochondria are dynamic organelles that undergo fission (mitochondrial fragmentation), fusion (tubular mitochondrial network), and transportation. Rapid regulation of mitochondrial dynamics occurs in response to infection, physiological stress, and metabolic demands. It has been previously reported that HBV infection causes mitochondrial damage and oxidative stress [3–5]. The expression of HBx disrupts mitochondrial respiratory complexes I, III, and IV, leading to reduced ΔΨm and antioxidant capacities, and increasing the formation of mtROS [44]. A previous study has demonstrated that the effect of HBx on mitochondrial function is genotype-dependent, with genotype D exerting an intermediate impact among all genotypes [45]. Consistently, all HBV models used in our study were derived from genotype D. Given that HBx markedly inhibits mitochondrial respiratory chain complex I in the present study, Rot was employed to pharmacologically mimic the mitochondrial suppression induced by HBx.

Inhibition of mitochondrial respiratory chain complex I leads to excessive production of ROS, particularly mtROS, which act as key mediators of oxidative stress. mtROS oxidatively damages the integrity of the lysosomal membrane, which is important for the fate of cells. Once LMP deteriorates, various hydrolases are released into the cytoplasm, typically resulting in the initiation of apoptotic cascades or other programmed cell death pathways [46–49]. HBV may thus finely balance these effects: sufficiently disrupting lysosomal integrity to block lysosomal degradation while avoiding excessive mitochondrial damage that would trigger apoptosis and eliminate viral replication, ultimately facilitating persistent infection. Future studies should delineate how HBV coordinates these pathways to sustain persistent infection.

Compared with mtROS-induced LMP, our understanding of the regulation of lysosomes by mitochondrial ATP remains limited. Mitochondria and lysosomes are essential for maintaining cellular homeostasis, and dysfunction of these organelles has been observed in various chronic diseases [50,51]. Lysosomes participate in multiple quality control mechanisms in mitochondria. Misfolded mitochondrial proteins or damaged mitochondria are transported to lysosomes for degradation through mitochondria-derived compartments (MDCs), mitochondria-derived vesicles (MDVs), and mitophagy [52]. Direct physical contact between lysosomes and mitochondria contributes to mitochondrial fission and homeostasis [10]. Conversely, disruption of the mitochondrial respiratory chain significantly inhibited lysosomal activity. Deletion of the mitochondrial respiratory chain leads to AMP-activated protein kinase (AMPK) inactivation, which inhibits lysosomal function [12]. Additionally, mitochondrial respiration deficiency indicates an imbalance in the NAD^+^/NADH ratio, whereas restoring NAD^+^ levels improves lysosomal function [11]. Notably, the glycolytic enzymes glyceraldehyde-3-phosphate dehydrogenase (GAPDH) and phosphoglycerate kinase 1 (PGK1) are localized to the lysosomal membrane. Mitochondrial translation deficiency reduces NAD^+^ synthesis, thereby impairing PGK1-catalyzed ATP production and affecting lysosomal acidity [53]. Thus, the question arises as to which ATP source, mitochondrial oxidative phosphorylation or glycolysis, is more critical for maintaining lysosomal acidity. Our findings demonstrate that ATP produced by the mitochondria is essential for v-ATPase assembly and lysosomal acidity. Although Rot/OA inhibited mitochondrial ATP production, increased glycolysis did not rescue lysosomal acidity. Interestingly, intracellular positioning of lysosomes dictates luminal pH [54]. Lysosomes are more acidic in the perinuclear region, where the ER-lysosome-mitochondrion three-way contact occurs, indirectly underscoring the importance of mitochondria in maintaining lysosomal acidity under normal physiological conditions.

Furthermore, mitochondrial damage hinders assembly of the V1 region with the V0 region of v-ATPase, leading to impaired lysosomal v-ATPase function. Mitochondrial ATP and the contacts between mitochondria and lysosomes are crucial for regulating v-ATPase assembly. However, the reasons why disruption of the mitochondrial respiratory chain affects mitochondria-lysosome contacts, and why the contact between mitochondria and lysosomes influences the assembly of v-ATPase, remain unclear. We speculate that when mitochondria interact with lysosomes, the higher ATP concentration in the mitochondria provides a favourable environment for triggering v-ATPase assembly. This process provides the necessary energy for v-ATPase to pump H^+^ into lysosomes, thereby maintaining the lysosomal pH. When mitochondrial ATP production is inhibited, the duration of mitochondria-lysosome contacts is reduced, as lysosomes are unable to obtain ATP from mitochondria.

In addition to CHB, mitochondrial and lysosomal co-inactivation occurs in various noninfectious chronic diseases, including neurodegenerative disorders and aging. However, the contribution of mitochondrial and lysosomal dysfunctions to disease chronicity remains poorly understood. Notably, in chronic HBV infection, lysosomal inactivation impedes HBV autophagic degradation, facilitating egress of HBV particles via the autophagic secretory pathway [14]. In parallel, mitochondrial dysfunction enhances HBV gene expression in HBV-infected hepatocytes [55], which was also observed in the present study. The mitochondrial-derived short peptide MOTS-c, known for enhancing mitochondrial function, inhibits HBV replication in the liver of HBV-infected mice and cells [56]. Both mitochondria and lysosomes serve as metabolic hubs within cells, and disruptions in metabolic homeostasis may significantly affect the viral life cycle and the immune microenvironment. Remarkably, mitochondria play a key role in innate immunity and are disrupted by HBV infection [57]. Additionally, lactate production contributes to HBV evasion by host innate immune surveillance [58]. Investigating the interplay between these metabolic imbalances and HBV infection may yield valuable insights into disease chronicity.

In conclusion, our findings suggest that HBV infection inhibits cellular autophagic degradation by regulating mitochondria- dependent LMP and v-ATPase assembly. These findings help to better understand the molecular mechanisms of HBV infection and provide a theoretical basis for the development of new antiviral treatment strategies.

## Author contributions

ZQ. W performed the experiments and prepared the manuscript; YY. Y, LZ. Z, C. L, JJ. Q, DD. C, XZ. H, MW. L, and GH. L helped with the experiments; ZY. X and ZG. Y helped with data analyses and graphics layout; XY. W and M. Z supervised the overall project, contributed to the development of the experimental plan, and revised the manuscript.

## Supplementary Material

Supporting information revised.docx

## Abbreviations

**Abbreviations**: ATP: adenosine triphosphate; CHB: chronic hepatitis B; CTSD: Cathepsin D; CTSL: Cathepsin L; DCFH-DA: 2’: 7’-dichlorofluorescin diacetate; ER: endoplasmic reticulum; HBV: Hepatitis B virus; HBV RIs: HBV replication intermediates; HBcAg: HBV core protein; HBsAg: HBV surface antigen; LC3: microtubule-associated protein 1 light chain 3; LAMP1: lysosomal-associated membrane protein 1; LMP: lysosomal membrane permeabilization; MVB: multivesicular body; SQSTM1: sequestosome-1; PHH: primary human hepatocytes; ROS: reactive oxygen species; SOD2: superoxide dismutase 2; TFEB: transcription factor EB; TMRM: tetramethyl rhodamine methyl ester; TOMM20: translocase of outer mitochondrial membrane 20; v-ATPase: vacuolar (H+)-adenosine triphosphatase; VPS13A: vacuolar protein sorting 13 A; SEM: standard error of the mean.
