# Supplementary material for: Mitochondrial respiratory chain regulates HBV clearance through dual modulation of lysosomal acidification
Source: Emerg Microbes Infect. 2025 Oct 7;14(1):2563079. doi: 10.1080/22221751.2025.2563079 (PMC12507116; doi:10.1080/22221751.2025.2563079)
Supplement: Supporting information revised.docx [file TEMI_A_2563079_SM3306.docx]

**Mitochondrial Respiratory Chain Regulates HBV Clearance through Dual Modulation of Lysosomal Acidification**

Zhiqiang Wei^1^, Yanying Yan^1^, Lingzhu Zhao^1^, Chen Li^1^, Jinjin Qi^1^, Dandan Chen^1^, Xiuzhen Huang^1^, Minwei Li^1^, Zhengyun Xiao^1^, Guohua Lou^1^, Zhenggang Yang^1^, Mengji Lu^2^, Xueyu Wang^1, *^, Min Zheng^1, *^

1. State Key Laboratory for Diagnosis and Treatment of Infectious Diseases, National Clinical Research Center for Infectious Diseases, Collaborative Innovation Center for Diagnosis and Treatment of Infectious Diseases, The First Affiliated Hospital, College of Medicine, Zhejiang University, Hangzhou, 310003, China

2. Institute of Virology, University Hospital Essen, University of Duisburg-Essen, Essen 45122, Germany

**Supplement Figures and tables**

**
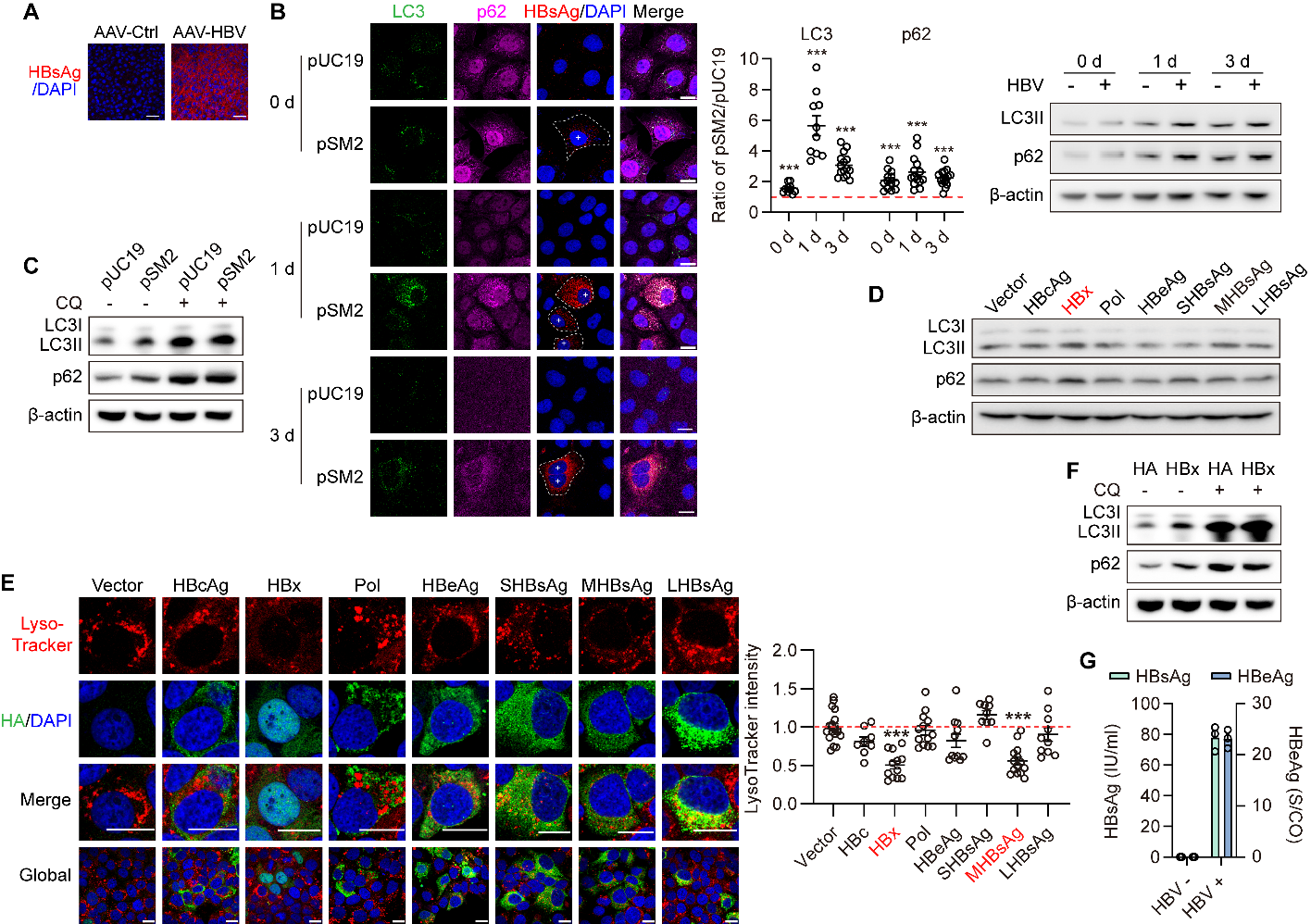
**

**Figure S1. HBV gene expression blocks lysosomal activation.** (A) Male C57BL/6 mouse (8 weeks old) were transduced with AAV-HBV1.2 through tail vein injection for 4 weeks. Liver tissues were stained with HBsAg, and then detected by IF. (B) Huh7 cells were transiently transfected with pUC19 or with pSM2 for 2 days or the indicated times. The expression of LC3 and p62 were measured by immunofluorescence and Western blotting. Scale bar, 20 μm. (C) Huh7 cells were transiently transfected with pUC19 or with pSM2, with or without CQ treatment for 2 days. The expression of LC3 and p62 were measured by Western blotting. (D, E) Huh7 cells were transfected with HA-HBcAg, HA-HBx, HA-Pol, HA-HBeAg, HA-SHBsAg, HA-MHBsAg, HA-LHBsAg and empty vector for 2 days. (D) The expression of LC3 and p62 were measured by Western blotting. (E) The cells were stained with 50 nM LysoTracker Red for 2 h. The expression of HA-tag was measured by immunofluorescence. The fluorescence intensity of LysoTracker Red was analyzed. Scale bar, 20 μm. (F) Huh7 cells were transfected with HA-HBcAg, HA-HBx, combined with or without CQ treatment for 2 days. The expression of LC3 and p62 were measured by Western blotting. (G) PHH cells were infected with HBV particles for 5 days. The levels of secreted HBsAg and HBeAg were measured. **p* <0.05; ***p* <0.01; ****p* <0.001; ns, not significant.

**
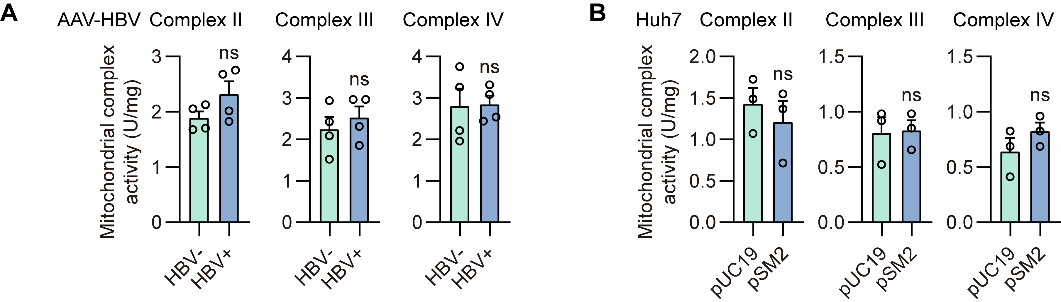
Figure S2. HBV gene expression does not inhibit mitochondrial respiratory chain complexes II-IV in vitro and in vivo.** (A) Male C57BL/6 mouse (8 weeks old) were transduced with AAV-HBV1.2 through tail vein injection for 4 weeks. (B) Huh7 cells were transiently transfected with pUC19 or with pSM2. Mitochondria were isolated from cell lysis or liver tissues to measure the activity of mitochondrial respiratory chain complexes II-IV. ns, not significant.


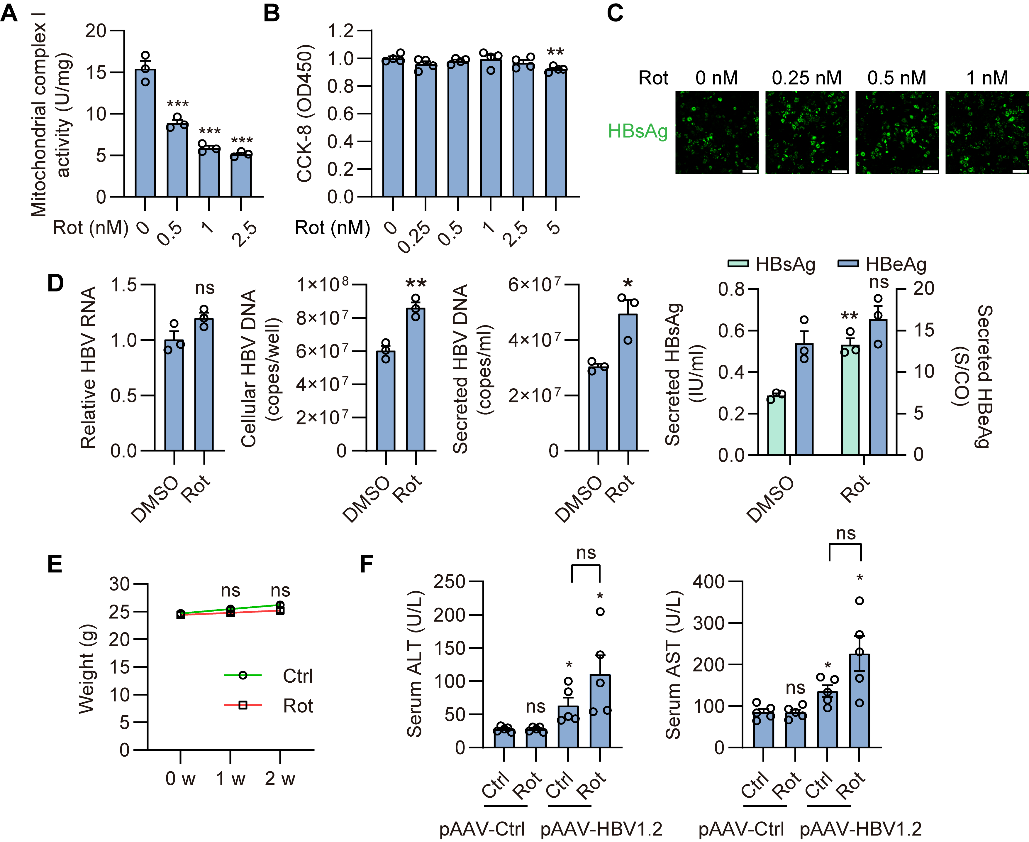


**Figure S3. Rot enhances HBV production.**  (A-C) Huh7 cells were transiently transfected with HBV construct plasmid (pSM2) for 6 h, then treated with indicated concentration of Rot for 2 or 3 days. (A) Mitochondria were isolated from cell lysis or liver tissues to measure the activity of mitochondrial respiratory chain complexes I. (B) Cell viability was detected using a Cell Counting Kit-8 (CCK-8) Assay kit. (C) The distribution of HBsAg were imaged by confocal immunofluorescence microscopy. Scale bar, 200 μm. (D) NTCP-HepG2 cells were infected with HBV particles for 24 h, and then the cells were treated with indicated concentration of Rot for 4 days. HBV RNAs were extracted and quantified by RT-qPCR. Intracellular HBV DNA levels were measured by qPCR. The levels of HBsAg, HBeAg, and HBV DNA in the culture supernatants were determined using qPCR and CMIA, respectively. (E, F) C57BL/6 mice (n = 26) received hydrodynamic injection (HDI) with 10 μg of pAAV-HBV1.2 plasmid. 1 week after HDI for, the mice were treated with CMC-Na (Ctrl, n = 13) or 8 mg/kg Rot (n = 13) by daily oral gavage for 2 weeks. (E) The weight was measured at 0, 1 and 2 weeks after CMC-Na or Rot treatment. (F) The levels of ALT and AST were detected. **p* <0.05; ***p* <0.01; ****p* <0.001; ns, not significant.

**
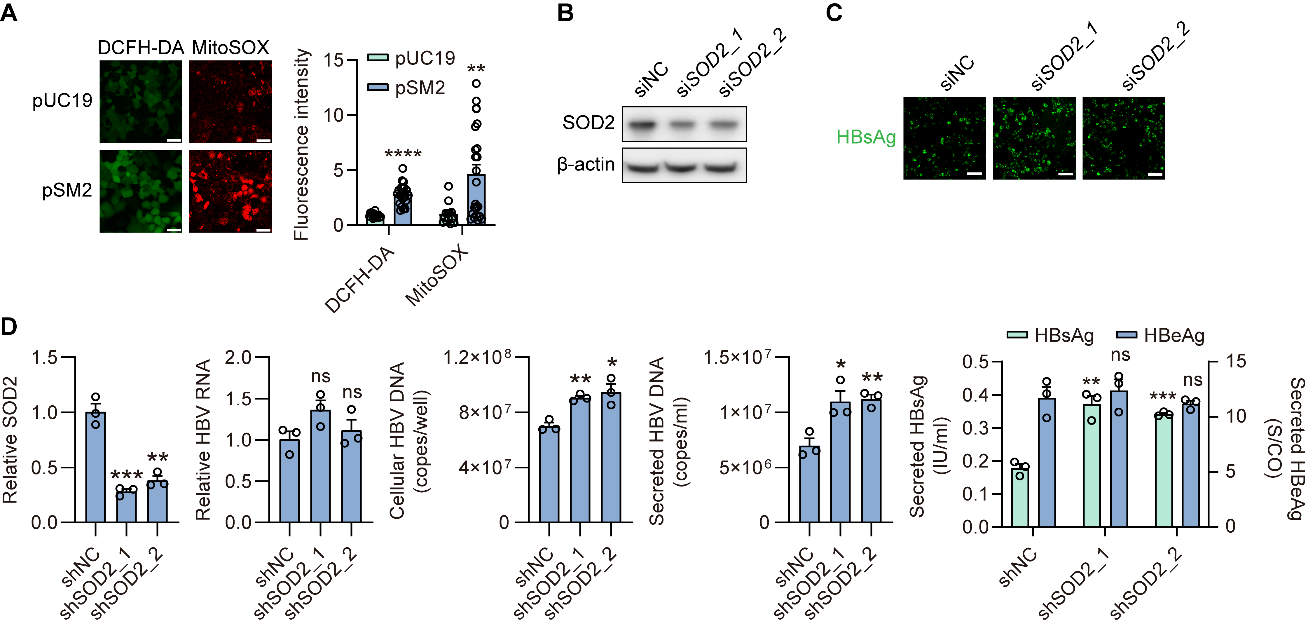
**

**Figure S4.** **HBV gene expression induces ROS, and SOD2 knockdown promotes HBV production in hepatoma cells lines.** (A) Huh7 cells were transiently transfected with pUC19 or with pSM2 for 2 days. DCFH-DA and mitoSOX staining were used to detect total ROS levels and mtROS levels, respectively. Scale bar, 50 μm. (B, C) Huh7 cells were transiently cotransfected with 40 nM siSOD2_1, siSOD2_2 or negative control siRNA (siNC), and HBV construct plasmid (pSM2) for 2 days. (B) The expression of SOD2 was measured by Western blotting. (C) The distribution of HBsAg were imaged by confocal immunofluorescence microscopy. Scale bar, 200 μm. (D) NTCP-HepG2 cells were infected with HBV particles for 24 h, followed by transduction with shSOD2 or shNC for an additional 24 h. The culture was subsequently maintained for a total of 4 days. HBV RNAs were extracted and quantified by RT-qPCR. Intracellular HBV DNA levels were measured by qPCR. The levels of HBsAg, HBeAg, and HBV DNA in the culture supernatants were determined using qPCR and CMIA, respectively. **p* <0.05, ***p* <0.01; ****p* <0.001.


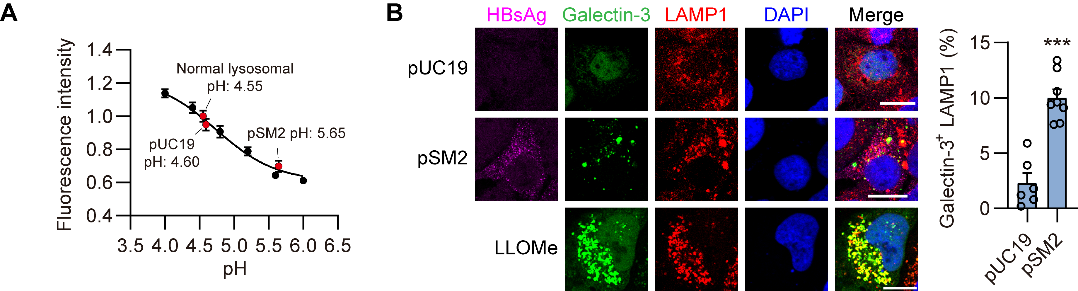
**Figure S5. HBV gene expression leads to increased pH and LMP.** (A) Huh7 cells were transiently transfected with pSM2. The cells were stained with 1 µM LysoSensor Green DND-189 for 1 h and analyzed by confocal microscopy. The fluorescence intensity was converted into lysosomal pH according to the standard curve. (B)The cells were cotransfected with pUC19 or with pSM2, and Galectin 3-EGFP plasmid. Colocalization of Galectin 3 and LAMP1 were analyzed. L-Leucyl-L-leucine methyl ester (LLOMe), the best characterized lysosomotropic agent that polymerizes inside lysosomes to induce fast but reversible lysosomal membrane damage, was used as a positive control.

**
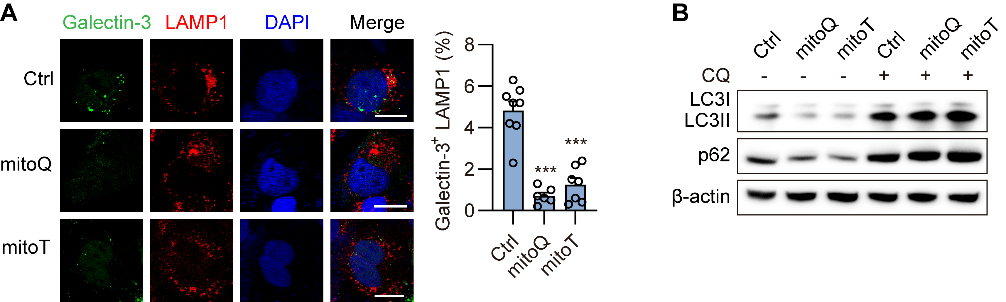
**

**Figure S6.** **Scavenging of mitochondrial ROS mitigates LMP and rescues autophagic flux.** Huh7 cells were transiently transfected with HBV construct plasmid pSM2, then treated with indicated concentration of mitoQ or mitoT for 2 days. (A) The cells were cotransfected with pUC19 or with pSM2, and Galectin 3-EGFP plasmid. Colocalization of Galectin 3 and LAMP1 were analyzed. (B) The cells were cotreated with CQ. The expression of LC3 and p62 were measured by Western blotting.

**
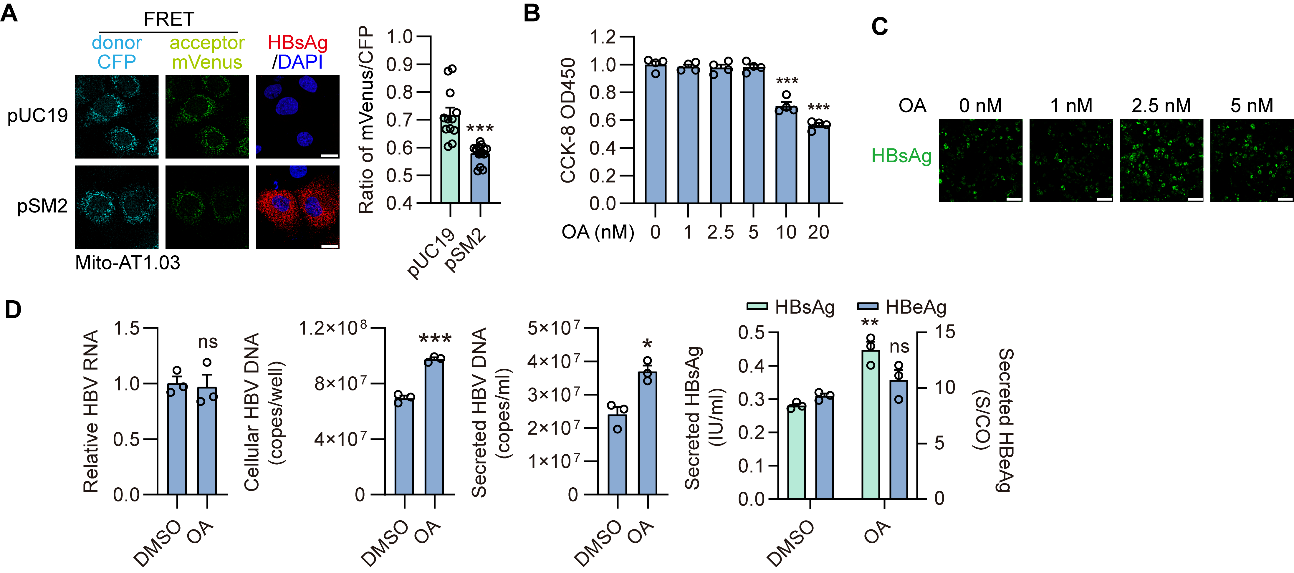
**

**Figure S7. HBV gene expression reduces mitochondria ATP production, and OA treatment promotes HBV production in hepatoma cell lines.** (A) Huh7 cells were transiently co-transfected with empty vector (pUC19) or HBV construct plasmid pSM2 and Mito-AT1.03 plasmid for 2 days. Mitochondria-specific ATP production was detected as described. (B, C) Huh7 cells were transiently transfected with HBV construct plasmid (pSM2), 6 hours post transfection, the indicated concentration of OA was added into the cell culture for 2 days. (B) Cell viability was detected using a Cell Counting Kit-8 (CCK-8) Assay kit. (C) The distribution of HBsAg were imaged by confocal immunofluorescence microscopy. Scale bar, 200 μm. (D) NTCP-HepG2 cells were infected with HBV particles for 24 h, and then the cells were treated with indicated concentration of OA for 4 days. HBV RNAs were extracted and quantified by RT-qPCR. Intracellular HBV DNA levels were measured by qPCR. The levels of HBsAg, HBeAg, and HBV DNA in the culture supernatants were determined using qPCR and CMIA, respectively. **p* <0.05; ***p* <0.01; ****p* <0.001; ns, not significant.


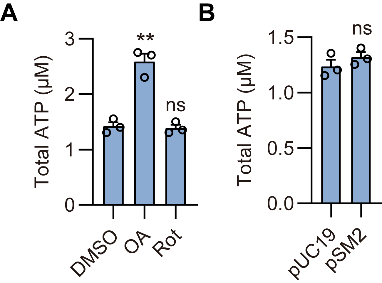


**Figure S8. Inhibition of mitochondrial function or ATP production, without a decrease in total ATP levels, was observed in hepatoma cell lines.** (A）Huh7 cells were treated with OA or Rot for 2 days. (B) Huh7 cells were transiently transfected with pUC19 or with pSM2 for 2 days. (A, B) Total ATP levels in intracellular were measured.

**
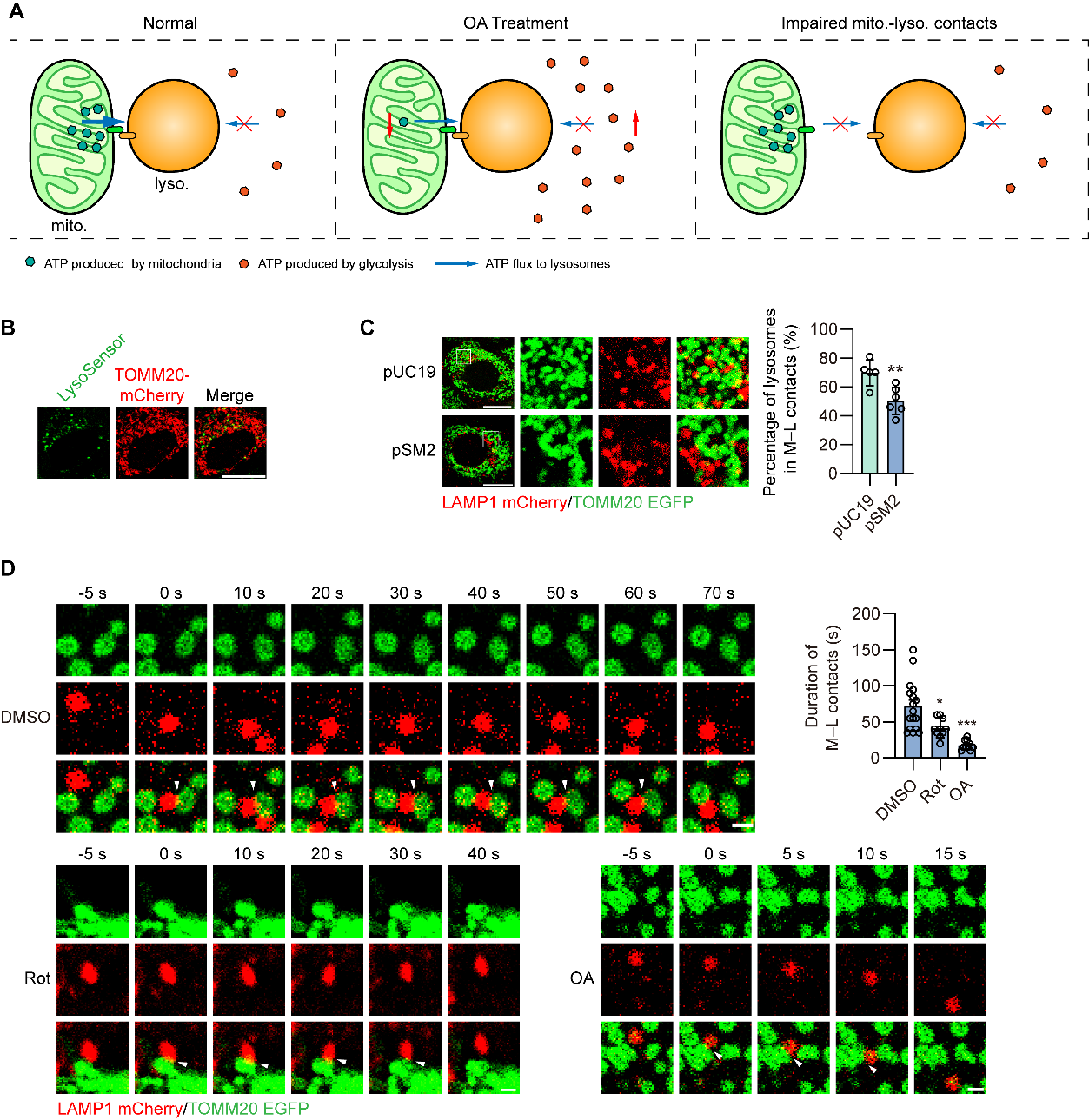
Figure S9. Inhibition of mitochondrial function decreased mitochondria-lysosome contacts.** (A) Schematic representation of the hypothesis that mitochondrial ATP production flux to lysosomes via mitochondria-lysosome physical contacts. (B) Huh7 cells were transiently transfected with TOMM20-mCherry plasmid for 2 days. Cells were stained with 1 µM LysoSensor Green DND-189 for 1 h. The fluorescence of LysoSensor and TOMM20-mCherry were imaged by confocal microscopy. Scale bar, 20 μm. (C) The Huh7 cell expressing TOMM20-EGFP, LAMP1-mCherry and HBV construct plasmid (pSM2). The percentage of lysosomes in mitochondria-lysosome contacts (M-L contacts (%)) were detected. (D) Huh7 cell expressing TOMM20-EGFP, LAMP1-mCherry, and then treat with Rot or OA. Quantitation of duration of mitochondria-lysosome contacts within the cytosol from time lapse images. **p* <0.05; ***p* <0.01; ****p* <0.001; ns, not significant.

**
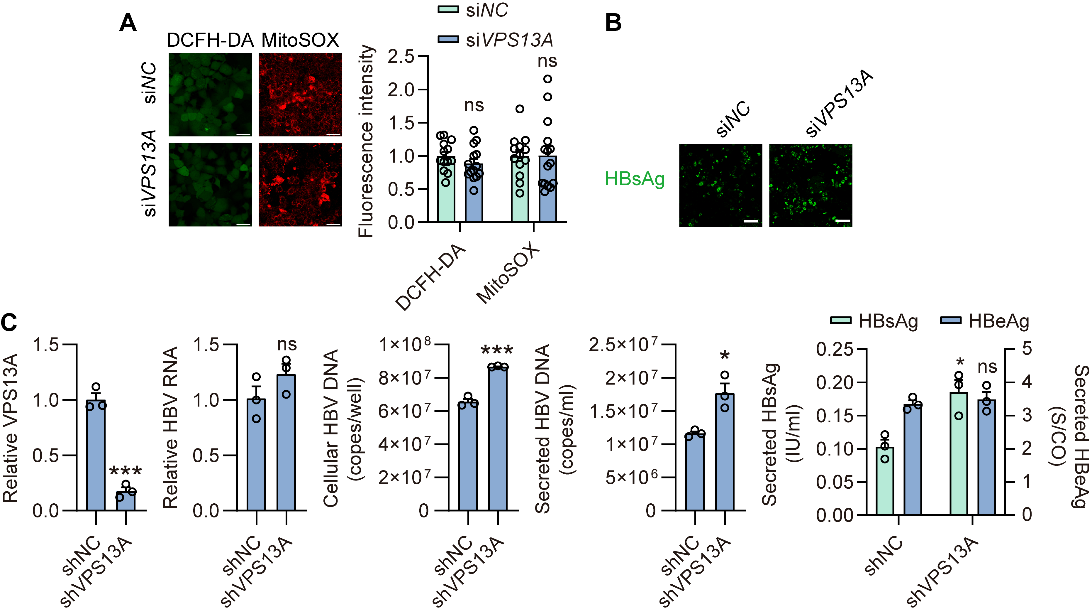
**

**Figure S10. Knockdown VPS13A decreases mitochondria-lysosome contacts, and enhances HBV production in hepatoma cell lines, without induce ROS.** (A, B) Huh7 cells were transiently cotransfected with 40 nM siVPS13A or siNC and HBV construct plasmid (pSM2) for 2 days. (A) DCFH-DA and mitoSOX staining were used to detect total ROS levels and mtROS levels, respectively. Scale bar, 50 μm. (B) The distribution of HBsAg were imaged by confocal immunofluorescence microscopy. Scale bar, 200 μm. (C) NTCP-HepG2 cells were infected with HBV particles for 24 h, followed by transduction with shVPS13A or shNC for an additional 24 h. The culture was subsequently maintained for a total of 4 days. HBV RNAs were extracted and quantified by RT-qPCR. Intracellular HBV DNA levels were measured by qPCR. The levels of HBsAg, HBeAg, and HBV DNA in the culture supernatants were determined using qPCR and CMIA, respectively. **p* <0.05; ***p* <0.01; ****p* <0.001; ns, not significant.

**
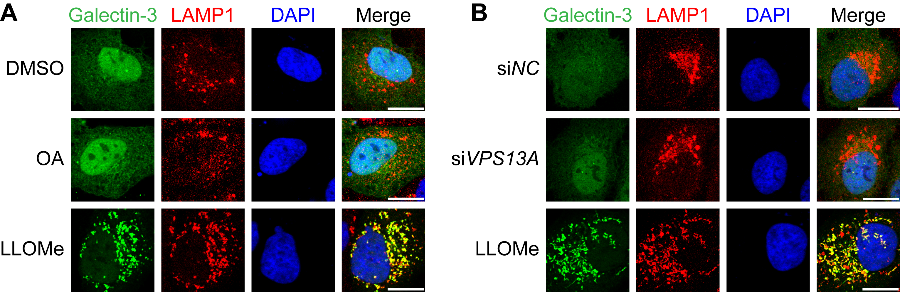
**

**Figure S11. OA treatment or VPS13A knockdown have no effect on LMP.** (A, B) The cells were cotransfected with HBV replication plasmid pSM2 and Galectin 3-EGFP plasmid. (A) Cells were treated with 5 nM OA for 2 days. (B) Cells were additionally transfected with siNC or siVPS13A. Colocalization of Galectin 3 and LAMP1 were analyzed by confocal immunofluorescence microscopy. Scale bar, 20 μm. LLOMe was used as a positive control.

**
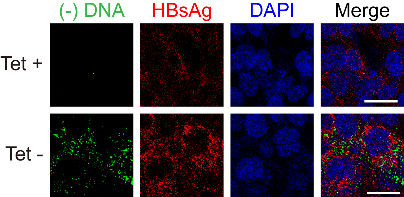
**

**Figure S12. Specific detection of HBV DNA and HBsAg by FISH in HepAD38 cells.** HepAD38 cells were cultured under Tet-regulated conditions to control HBV replication. Cells maintained in the presence of Tet (+) suppress HBV replication, whereas removal of Tet (–) induces viral transcription and particle production. After 4 days of culture, cells were subjected to RNAscope fluorescence in situ hybridization (FISH) using a probe targeting the single-stranded region of HBV rcDNA. Fluorescence signals were visualized by confocal microscopy.

**Table S1.** List of siRNA/shRNA

| Target | Target Sequence (5′-3′) |
| --- | --- |
| SOD2_1 | AAGTAAACCACGATCGTTA |
| SOD2_2 | CAACAGGCCTTATTCCACT |
| VPS13A_1 | GCAGCTACATTCCTCTTAA |
| VPS13A_2 | GGATAGAGCTTATGATTCA |

**Table S2.** List of Plasmids

| Plasmid | Source |
| --- | --- |
| pLAMP1-mCherry | Addgene, 45147 |
| pFX-TOMM20-EGFP | Miaoling Biology, P47867 |
| mCherry-TOMM20-N-10 | Addgene, 55146 |
| pCMV-Mito-AT1.03 | Beyotime Biotechnology, D2606 |
| pECMV-3×FLAG-SOD2 | Miaoling Biology, P4127 |
| pEGFP-hGal3 | Addgene, 73080 |
| pEnCMV-TFEB-3×FLAG | Miaoling Biology, P20646 |

**Table S3.** List of Reagents

| Reagent | Company |
| --- | --- |
| LC3B (E5Q2K) Mouse mAb | Cell Signaling Technology, 83506 |
| LC3B (D11) XP Rabbit mAb | Cell Signaling Technology, 3868 |
| SQSTM1/p62 Antibody | Cell Signaling Technology, 5114 |
| SQSTM1/p62 (D5L7G) Mouse mAb | Cell Signaling Technology, 88588 |
| beta Actin Mouse mAb | Santa Cruz Biotechnology, sc-47778 |
| Mitochondrial Dynamics Antibody Sampler Kit II | Cell Signaling Technology, 74792 |
| Parkin Antibody (1H4) Mouse mAb | Novus Biologicals, H00005071-M01 |
| HBsAg Mouse Antibody | ZAGB-BIO, ZM-0122 |
| HBsAg Horse Antibody | Abcam, ab9193 |
| HBcAg Rabbit Antibody | Abcam, ab115992 |
| HA-Tag (C29F4) Rabbit mAb | Cell Signaling Technology, 3724 |
| SOD2 (D3X8F) XP Rabbit mAb | Cell Signaling Technology, 13141 |
| LAMP1 (D2D11) XP Rabbit mAb | Cell Signaling Technology, 9091 |
| LAMP1 (D4O1S) Mouse mAb | Cell Signaling Technology, 15665 |
| Cathepsin B Rabbit Antibody | Cell Signaling Technology, 31718 |
| Cathepsin D Rabbit Antibody | Cell Signaling Technology, 2284 |
| Cathepsin L Rabbit Antibody | Cell Signaling Technology, 71298 |
| Chorein/VPS13A Antibody | Abcam, ab65787 |
| ATP6V0D1/P39 antibody | Abcam, ab202897 |
| ATP6V1A antibody | Abcam, ab199326 |
| ATP6V1D antibody | Abcam, ab157458 |
| mTOR (7C10) Rabbit mAb | Cell Signaling Technology, 2983 |
| Phospho-mTOR (Ser2448) (D9C2) XP Rabbit mAb | Cell Signaling Technology, 5536 |
| LysoTracker Red DND-99 | Thermo Fisher Scientific, L7528 |
| MitoTracker Deep Red FM | Thermo Fisher Scientific, M22426 |
| DAPI Staining Solution | Beyotime Biotechnology, C1005 |
| Image-iT TMRM Reagent | Thermo Fisher Scientific, I34361 |
| Seahorse XF Cell Mito Stress Test Kit | Agilent Technologies, 103015-100 |
| DCFH-DA | Beyotime Biotechnology, S0033 |
| mitoSOX | Thermo Fisher Scientific, M36009 |
| Rotenone | Sigma-Aldrich, 45656 |
| DIG-High Prime DNA Labeling and Detection Starter Kit II | Roche, 11585614910 |
| TB Green Premix Ex Taq II | Takara Bio, RR820A |
| One Step TB Green PrimeScript RT-PCR Kit II | Takara Bio, RR086A |
| ARCHITECT HBsAg Reagent Kits | Abbott |
| ARCHITECT HBeAg Reagent Kits | Abbott |
| TIANamp Virus DNA/RNA Kit | TIANGEN, DP315 |
| LysoSensor Green DND-189 | Thermo Fisher Scientific, L7535 |
| Sodium Monensin (NSC 343257) | Selleckchem, S2324 |
| Nigericin sodium salt | Selleckchem, S6653 |
| L-Leucyl-L-Leucine methyl ester hydrochloride /LLOMe | MedchemExpress, HY-129905 |
| Mitoquinone (MitoQ10) mesylate/ | Selleckchem, S8978 |
| Mito-TEMPO | Selleckchem, S9733 |
| Oligomycin A | Selleckchem, S1478 |
| Cell Counting Kit-8 | Beyotime Biotechnology, C0038 |
| ATP Assay Kit | Beyotime Biotechnology, S0026 |
| Mitochondrial complex I/NADH-CoQ reductase Activity Assay Kit | Solarbio, BC0515 |
| Mitochondrial complex II/succinate-coenzyme Q reductase Activity Assay Kit | Solarbio, BC3235 |
| Mitochondrial complex III / CoQ-cytochrome C reductase Activity Assay Kit | Solarbio, BC3245 |
| Mitochondrial Complex IV / Cytochrome C Oxidase Activity Assay Kit | Solarbio, BC0945 |
| RNAscope® Probe- V-HBVGTD-sense | ACDBio, 448791 |
| RNAscope Multiplex Fluorescent Reagent Kit v2 | ACDBio, 323100 |

**Table S4.** List of Primers

| Gene | Sequence (5′-3′) |
| --- | --- |
| HBV DNA | F: GTTGCCCGTTTGTCCTCTAATTC |
|  | R: GGAGGGATACATAGAGGTTCCTT |
| HBV RNA | F: CCGTCTGTGCCTTCTCATCT |
|  | R: TAATCTCCTCCCCCAACTCC |
